# Open source IoT-based collection bin applied to local plastic recycling

**DOI:** 10.1016/j.ohx.2022.e00389

**Published:** 2022-12-21

**Authors:** Alex Gabriel, Fabio Cruz

**Affiliations:** Université de Lorraine – ERPI – F-54000, Nancy, France

**Keywords:** Distributed recycling, IoT, Smart waste management

## Abstract

Plastic waste is a major challenge for policy making; it has a terrible impact on the environment if it is not properly managed. In order to mitigate this issue, recycling industries have emerged with the associated logistics chain that also has an environmental impact, notably with the production of greenhouse gas. In addition to using energy to transform plastic waste into source material, energy is also wasted to transport it. In parallel to reducing plastic waste, it may be recycled at a very local scale, reducing transportation and allowing potential improvement of the collecting process. Assuming that local transformation of plastic waste is possible, this article describes the design, assembly, and setup of the hardware, system architecture, and software of collectors that may be used by these recycling units. The specificity of these collectors is that they produces on-line data related to the quantity of waste collected. Once implemented, a network of smart collectors should allow the reduction of travel to collect waste as it notifies when the collectors are full. It also produces data on the scale of a territory to optimize the supply chain related to plastic waste collection. This article presents the design and engineering aspects as well as limitations induced by technical choices, but also potential improvements for future developments.

## Graphical abstract

**Figure:**
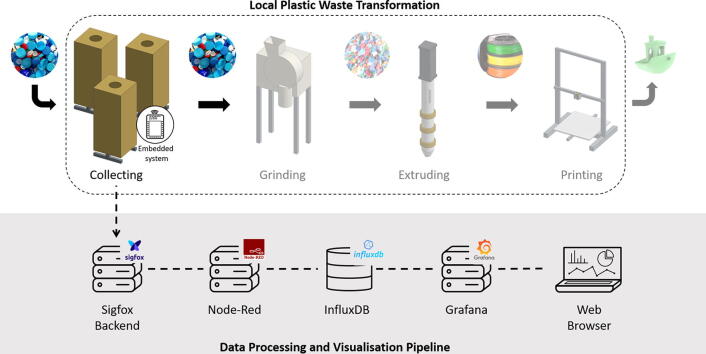

## 1 Specifications table

.

**Table:** 

**Hardware name**	*Open Source Smart Collector*
**Subject area**	• *Engineering and material science* • *Educational tools and open source alternatives to existing infrastructure for recycling*
**Hardware type**	• *Field measurements and sensors* • *Smart waste management*
**Closest commercial analog**	*Quamtra Smart Waste Management - Q sensor and Q mini*
**Open source license**	*Creative Commons Attribution 4.0 International License*
**Cost of hardware**	*Around €200*
**Source file repository**	http://doi.org/10.17605/OSF.IO/K9GRA

## 2 Hardware in context

Plastic waste is a major problem in policymaking and societal agendas given the fact that it is estimated that only 9% of total produced plastic has been recycled since 1950 [1]. The fundamental issue is that this material does not reintegrate into one of the relatively well-known biogeochemical cycles of the elements of our ecosystems [2]. Even more problematic is the fact that plastic waste is expected to increase from 6.3 billion metric tons in 2015 to a projected 26 billion tons by 2050 [3]. However, under current recycling conditions, this implies that around 80% will be disposed of in landfills or in the natural environment (more precisely, an estimated 4 million to 12 billion tons of waste plastic are entering oceans annually). Moreover, a set of systemic complexities including geopolitical [4], technical and behavior make it difficult to tackle this problem in a systematic way. In fact, the recent pandemic situation adds more complexity given the paradox of single-use plastics [5].

Today, much of the expectation with the circular economy strategy relies on the technical framework to enable the increase in cascading loops for materials and products [6], [7]. Several tools and methods are being developed to enhance the performance of the reverse supply chain, making a secondary raw material market possible[8]. The cross-line of Industry 4.0 and circular economy is opening up fields such as smart waste management system options to improve the effectiveness of different materials, including plastic waste [9], using information technology tools with the advent of the Internet of Things (IoT) [10], [11]. In this sense, the development of technical IoT-based solutions seeks three main purposes:

• Improve the efficiency of waste collection and separation at the source
• Foster waste management activities and practices from recycling to move up to the top of the waste hierarchy
• Minimize the adverse environmental implications

One of the leverage points to tackle plastic waste relies on improving selective waste collection, which makes it so that less and less recyclable waste goes to landfills. It is crucial for as much collected waste as possible to be recycled [12]. Unfortunately, the individual’s behavior and lack of sufficient facilities are, among others, key factors that impact the high volume of plastic litter in the environment [13]. Extensive research has been undertaken to understand how a collective dynamic on a local scale can involve waste producers in recycling and taking part in a circular economy [14], [15]. In the context of this article, the perspective is to study the technical infrastructure to collect waste production data and properly design the logistics of the circular economy supply chain. This waste production data on a local scale is notably required for closed loop supply chains for circular economy purposes [16], [17].

### 2.1 Challenges associated with plastic collection

In the literature, there are two modalities of collection processes for solid waste management strategies [18]:

• Door-to-door collection: this is a technique which provides a periodic pickup service.
• Indirect collection: this is based on the use of containers or communal bins placed near markets, in apartment complexes, and in other appropriate locations where special vehicles can collect the solid waste.

The smart collector designed in this article is used for indirect collection as it will be a setup in buildings that receive the public, such as schools, malls, or concert halls. The objective of this smart collector is to gather data concerning the quantity of plastic that can be collected to be valorized in a local distributed recycling production [19], [20], [21]. The approach can be seen in two phases: 1) a monitoring phase in which the fullness levels of rubbish bins are constantly measured, and 2), a computation phase in which collected data is elaborated for the optimization of trash collection as suggested by Catania and Ventura [18]. This article focuses on the technical aspects related to the devices that collect waste and generate data.

### 2.2 Collector and internet of things

Table 1 summarizes several waste collectors that exploit the capacities of IoT, which were identified in the literature. Notably, some of the characteristics of the hardware include power supply, network connectivity, type of sensor, and application domain. This literature overview maps two trends associated with smart waste collectors: 1) connected containers and 2) interactive containers. The first trend aims to create waste containers that collect data from the perspective of optimizing the waste disposal chain such as [18]. The second trend aims to embed electronics in the bin in order to explore new interaction modalities or sorting features such as [22]. However, the connected containers presented in Table 1 do not provide sources to replicate the devices. The aim of this article is to document the design of a connected container. The main rationale for the development of this open hardware relies on two arguments: First, to facilitate the plastic recycling, it is imperative to identify potential plastics that are considered recyclable without potential contamination during their life cycle. Second, this hardware, in the long run, aims to develop a methodology for the operational research field, with the purpose of enabling data about granular plastic production and collection in neighborhoods. On the micro scale, it is possible to identify micro-value chains of plastic recycling.

**Table 1:** Technologies used and application areas of smart collectors.

**Reference**	**Power supply**	**Type of sensor**	**Type of network**	**Domain**
[23]	Battery	Weight	WIFI	Food waste
[6]	N/A	Level sensor, humidity sensor, and load sensor	WIFI	General waste
[24]	Battery	Ultrasonic distance sensor, camera, strain gauges, liquid gauges	GPRS	General waste
[18]	Battery (not explicit)	Proximity sensor, weight sensor	Zigbee	Solid waste
[22]	12 V solar panel	Image acquisition (OV 2640 sensor, ESP 32-CAM), proximity sensor (HC-SR501), photodetector (RPR220)	N/A	General waste

From this perspective of bins that generate data, there are various aspects that can be considered, such as network connectivity, complexity of the infrastructure to deploy devices in the field, the type of sensor used, and the power supply.

One of the key requirements is the network connectivity of the device in order to transmit the information collected. According to the technology used, the network and the architecture will be more or less complex. Three articles identified used short-range communication technologies such as WIFI [6], [23] or ZigBee [18]. These technologies imply the deployment of the gateway over the area, such as those on lampposts [18] to transfer the data to distant servers. The IoT-based smart garbage system (SGS) from Hong et al. [23] was composed of several smart garbage bins (SGBs), routers, and servers. The main drawback of this network technology is the investment and the effort required for experimentation. WIFI is a Wireless Local Area Network (WLAN) technology; alternatives are Wide Area Network (WAN) such as LoRa, Sigfox, NB-IoT, and LTE-M [25] networks that are more adapted to IoT. This is the case with Vicentini et al. [24] work that used GPRS (General Packet Radio Service), which is a cellular communication norm. It is a predecessor norm of NB-IoT and LTE-M. One of the advantages of the WAN technologies is the high degree of flexibility, allowing designers to experiment over a large area without dealing with network infrastructure management.

Although the containers may communicate, the nature of the message will depend on the phenomenon observed and the sensors used. However, the main goal of these sensors is to monitor the fullness of the bins [18], [6]. To do so, parameters such as weight and volume are those most used in domains including food-waste [24], [18], [23]. The use of strain gauges is commonly used to measure the weight variable, whereas proximity sensors were used to estimate the volume of waste in the bin. An alternative to a proximity sensor may be a camera with image processing such as CleanWings [24]. However, CleanWings was designed in 2009; most of the technologies are outdated. Similar features could be achieved with much smaller, low-power devices such as the ESP32-cam,which was used in the smart waste bin from Li et al. [22].

Finally, communication and data acquisition require power sources. From the examples of Table 1, they mainly rely on batteries, though one mentions the usage of photovoltaic cells. It implies the use of compliant devices with a battery as a power source.

This overview shows that similar projects require heavy infrastructure such as routers and a gateway to deploy in the territory. There is an avenue to simplify experimentation in this domain using common open source technology (hardware and software)[26]. It means the device will use a controller compatible with batteries and use WAN technology to avoid the deployment of routers for data acquisition. Although using various types of sensors allows us to achieve better results [18] by crossing data, the main indicator remains the weight.

## 3 Hardware description

**Fig. 1:**
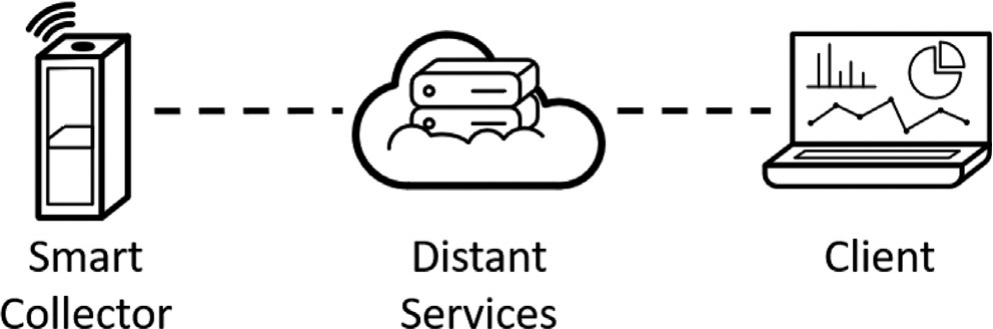
Basic principle schema of the smart collector system.

The smart collector is a technical device to collect discarded plastic where the main feature is to possibly measure the weight of its contents. The principle is to let several smart collectors in the neighborhood collect plastic and identify which quantity of plastic can be collected and when someone must pick the plastic up. To do so, the collector must communicate over a larger area than WIFI coverage. The communication solution must rely on Wide Area Network. The principle is that the smart collector sends the data on a distant server and this server provides a dashboard for people who must manage the plastic, as shown in Fig. 1. More specifically, this experimentation is based on Sigfox, which is the main distinction with the previous works.

The main functional requirement of the smart collector is to collect and provide data about plastic waste production in order to design a local and distributed recycling chain of value. However, the smart collector may be used in various use cases such as:

• Monitoring the quantity of any other product that is collected over a large area.
• Generating data about behavior to more precisely dimensions public infrastructure.
• Monitoring the transformation and recycling process inside the transformation unit to follow the state and quantity of raw material and final product.
• Initiating a digitization process in the waste management process as the information system element present here is flexible and commonly used in various types of projects.

## 4 Design files summary

Various alternatives to the smart collector have been realized, based on the microcontroller used. There are two versions of the electronic infrastructure: a LoPy4 and an ESP8266 version. In the present article, we will focus on the LoPy4 version. Nevertheless, there is the documentation of the ESP8266 version in the repository of the project. Also, the design files are common to both of these, such as the CAD files listed in Table 2.

**Table 2:** Design files

**Design file name**	**File type**	**Open source license**	**Location of the file**
Base Top	CAD file (DXF)	Creative Commons Attribution 4.0 International License	https://osf.io/5darv/
Base Bottom	CAD file (DXF)	Creative Commons Attribution 4.0 International License	https://osf.io/f4v3j/
Base Back	CAD file (DXF)	Creative Commons Attribution 4.0 International License	https://osf.io/xevnc/
Base Front	CAD file (DXF)	Creative Commons Attribution 4.0 International License	https://osf.io/rm7h5/
Base Spacer	CAD file (DXF)	Creative Commons Attribution 4.0 International License	https://osf.io/n7s3f/
Base HX711 Fastener	CAD file (DXF)	Creative Commons Attribution 4.0 International License	https://osf.io/xy67d/
Collector Front	CAD file (DXF)	Creative Commons Attribution 4.0 International License	https://osf.io/2y3w7/
Collector Back	CAD file (DXF)	Creative Commons Attribution 4.0 International License	https://osf.io/shbg2/
Collector Right	CAD file (DXF)	Creative Commons Attribution 4.0 International License	https://osf.io/6rqnt/
Collector Left	CAD file (DXF)	Creative Commons Attribution 4.0 International License	https://osf.io/239f7/
Collector Bottom	CAD file (DXF)	Creative Commons Attribution 4.0 International License	https://osf.io/t25ka/
Collector Top	CAD file (DXF)	Creative Commons Attribution 4.0 International License	https://osf.io/pmrfu/
Collector Fastener	CAD file (DXF)	Creative Commons Attribution 4.0 International License	https://osf.io/c4y9t/
Electric Schema LoPy4 ExpBoard	Image	Creative Commons Attribution 4.0 International License	https://osf.io/qmha7/
Electric Schema LoPy4 Shield	Image	Creative Commons Attribution 4.0 International License	https://osf.io/pkq7t/
Collector configuration	Spreadsheet	Creative Commons Attribution 4.0 International License	https://osf.io/95a6w/
Bill of Material - Lopy4	Spreadsheet	Creative Commons Attribution 4.0 International License	https://osf.io/w4abz/
Bill of Material - ESP8266	Spreadsheet	Creative Commons Attribution 4.0 International License	https://osf.io/sezqm
Firmware ESP8266	Program	Creative Commons Attribution 4.0 International License	https://osf.io/64y3p/
Firmware LoPy4	Program	Creative Commons Attribution 4.0 International License	https://osf.io/wrzpe/
Information System	Program	Creative Commons Attribution 4.0 International License	https://osf.io/9bws7/

**Fig. 2:**
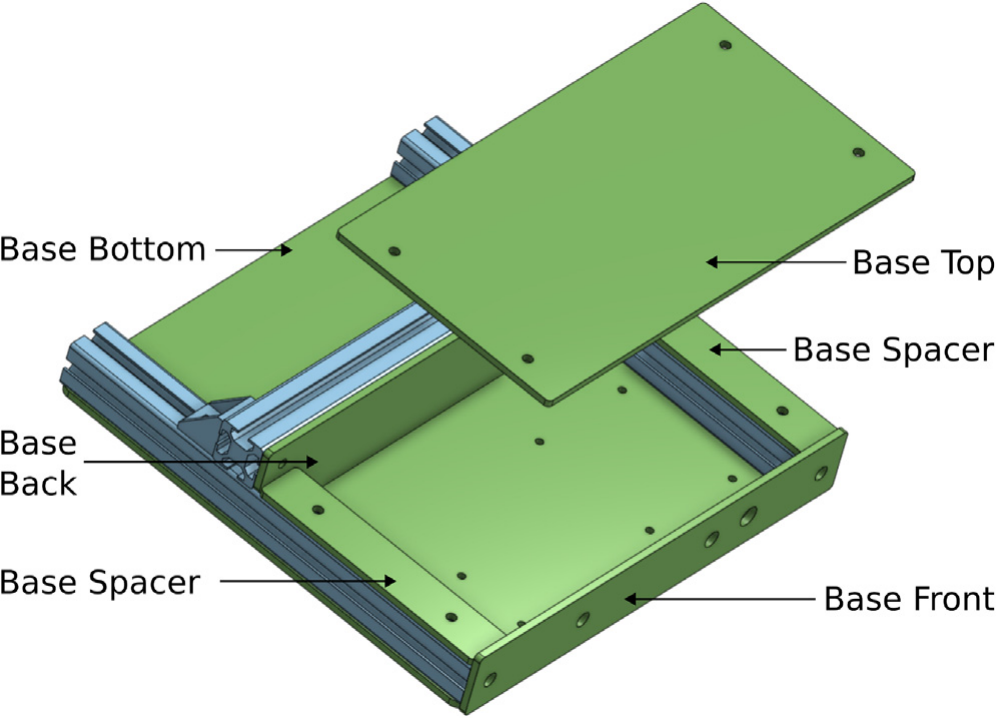
Position of the PMMA plate on the base of the smart collector.

The *Base Bottom*, *Base Top*, *Base Back*, and *Base Front* are PMMA plates which protect the electronics that will do the measuring and communicating. All these parts are laser-cut. The *Base Bottom* is the lowest PMMA plate, where the electronic components are fixed. This plate also stiffens the aluminum profile structure. The *Base Front* is a PMMA plate where the tare button and the power supply jack are fixed. The *Base Spacers* are two pieces used to adjust the height available inside for the electronic parts. These are optional. Fig. 2 illustrates how the *Base* parts are assembled with aluminum extrusion.

**Fig. 3:**
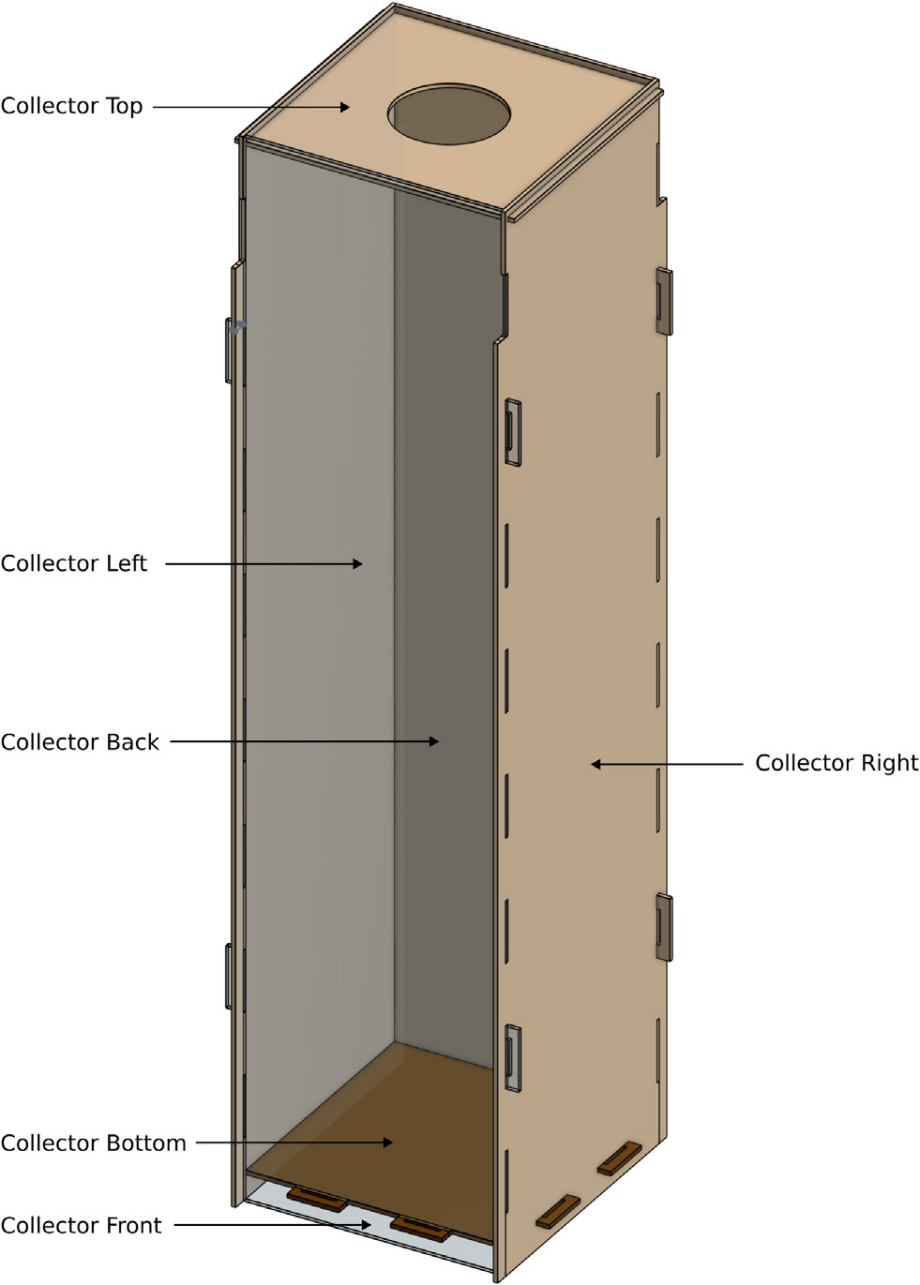
Elements of the collector.

The *Collector Front*, *Collector Back*, *Collector Left*, *Collector Right*, *Collector Top*, and *Collector Bottom* are the elements that form the receptacle where the plastic will be collected. These parts are also laser-cut. The *Collector Front* and *Collector Back* are identical as well as *Collector Left* and *Collector Right*. The only difference is the material chosen for the *Collector Front*; this material is transparent to allow people to see what there is inside. The other can be either PMMA or MDF. Fig. 3 shows what the collector looks like once assembled.

The Firmware Files contains two different firmware products according to the microcontroller used, depending on the network connectivity. It is either an Adafruit Huzzah ESP8266^1^ or a Pycom LoPy4 2 microcontroller. Both have been developed with IDE Visual Studio Code 3.

## 5 Bill of materials summary

The project was initially designed to use LoPy4 microcontrollers in order to utilize Sigfox LPWAN connectivity. However, difficulties in sourcing LoPy4 also led the team to develop a cheaper version based on ESP8266. The following bill of materials (Table 3) is for the LoPy4 version (also available at https://osf.io/w4abz/). The ESP8266 version of the bill of material is available at https://osf.io/sezqm/.

**Table 3:** Bill of material for the LoPy4 version of the smart collector.

**Designator**	**Component**	**Nb**	**Cost per unit – currency**	**Total cost – currency**	**Source of materials**	**Material type**
MCU	Pycom LoPy4	1	41.18€	41.18€	RS	Semi-conductor
EXT-ANT	Pycom LoRa-Sigfox Antenna	1	12.06€	12.06€	RS	Semi-conductor
MCU-DEVB	Pycom Expansion Board	1	22.94€	22.94€	RS	Semi-conductor
LOAD-CELL	Weight sensor (20 kg)	1	9.50€	9.50€	GoTronic	Semi-conductor
LOAD-AMP	HX711	1	4.30€	4.30€	GoTronic	Semi-conductor
BTN	Momentary Push button	1	2.93€	2.93€	RS	Metal Polymer
PWR-SP	5V 1A power supply	1	8.16€	8.16€	RS	Semi-conductor
PWR-CNT	DC socket 5A 12V pin diam. 2,5mm	1	8.17€	8.17€	RS	Metal
USB-ADPT	USB to cable adaptor	1	7.10€	7.10€	RS	Metal Polymer
BATTERY	LiPo battery 1.8Ah 3.7V	1	14.77€	14.77€	RS	Metal Polymer
JST-connector	JST connector	1	0.08€	0.08€	RS	Polymer
JST-contact	JST contact	2	0.02€	0.04€	RS	Metal
CHC-M4-8mm	CHC screw M4 – 8mm length	20	0.245€	4.90€	RS	Metal
TN-M4	T-nuts M4	26	1.175€	30.55€	RS	Metal
CHC-M3-20mm	CHC screw M3 – 20mm length	2	0.25€	0.50€	RS	Metal
Wire	electric wires for button and power supply	0.8	0.14€	0.11€	RS	Metal
TST-PLT	electric wires for button and power supply	1	1.20€	1.20€	GoTronic	Metal Polymer
SHLD-CNT	long connectors	1	0.70€	0.70€	GoTronic	Metal Polymer
PMMA-sheet1	PMMA sheet 400 mm × 500 mm for Base parts	1	22.39€	22.39€	RS	Polymer
PRF2020	Aluminum Bosch Profile 20 × 20 – 6 pieces of 200 mm	1200 mm	19.58€	11.748€	RS	Aluminum
BRK	Bracket Bosch Rexroth 6mm	8	3.68€	29.44€	RS	Aluminum

The total price of the prototype is not a reliable indicator for possible comparison with other solutions. The sourcing of materials is constrained by the policy of the university purchasing division. The component supplier has to be part of a market and their prices are not always the most competitive. A detailed bill of materials for the circuit board component is included in the design files along with the schematics and board.

The *MCU* has been chosen based on connectivity, versatility, and ease of deployment. The main constraint was the Sigfox connectivity. An alternative to the LoPy4^4^ would be the Arduino MKRFOX 1200^5^, which is less expensive as is it does not require any additional board to manage the battery. However, the benefit of the LoPy4 microcontroller is its embedding of multiple connectivity, notably LoRaWAN. Actually, the alternative to reduce the price is to change the LPWAN technology to LoRaWAN. Cheap microcontrollers based on ESP32 and embedding LoRa connectivity can be found on the internet. The main drawback is the network, which is more difficult to deploy and maintain. There are three scenarios for use of LoRaWAN:

• Using The Thing Network^6^, which is an open LoRaWan network. As it is a community-based network, not all the area is equally covered. In the current work, the experimentation area was not covered.
• Deploying a gateway to cover the local area. This can be done using Raspberry Pi, a LoRa shield, and the open source stack to manage the gateway ChirpStack^7^.
• Subscribing to operators to have access to industrial LoRaWAN.

The *MCU-DEVB* is embedded in the prototype as it facilitates the charge/discharge of a battery. For a cheaper prototype, this shield could be replaced by a battery management system based on a TP4056 chip.

The *BATTERY* is optional. It depends on the usage. Based on the connectivity of the *BATTERY*, it may be necessary to change it to the proper connector, corresponding to those on *MCU-DEVB*. It is for this reason that there are *JST-contact* and *JST-connector* in the bill of material. However, these two items are not necessary if you don’t use the battery.

## 6 Build instructions

As with every connected device, there are three main aspects to consider: the program of the device, the hardware of the device, and the information system that will handle the data of the device. This section will be structured in the opposite way, meaning the information system will be presented first because it will define variables required by the device program.

### 6.1 Information system

The choice of this project is to use a Pycom LoPy4 microcontroller and the Sigfox connectivity. Due to this, there are two possibilities to visualize the evolution of the sensor connected to the microcontroller:

• Using the Pybytes platform^8^ which makes it possible to monitor and manage the Pycom microcontroller remotly.
• Deploying an entire back-end based on a database such as InfluxDB^9^ and visualization tools such as Grafana^10^.

**Fig. 4:**
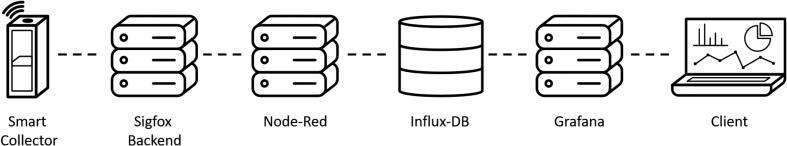
Architecture of the information system.

In order to control the lifetime of the data and have higher flexibility for data visualization, the second scenario has been preferred. The architecture of the information system is represented in Fig. 4.

Each component in Fig. 4 has a specific role described as follows:

• The Sigfox Backend is a hosted service managed by Sigfox. The device must be registered on the platform. Once registered, the data sent by the device are centralized on the platform. This data can be transferred to another platform through the Callback function.
• The Node-RED^11^ service is a block-based web IDE that allows for the creation of API. The role of this service is to receive the raw data from Sigfox Backend, parse it and save it in Influx DB.
• The InfluxDB is a database specialized in time series. The role of this service is to store the data concerning the different devices in order to visualize and analyze it.
• The Grafana service is a visualization tool that has to be connected to a database. In this experience, Grafana is connected to InfluxDB and enables the creation of a dashboard for each device that shows the weight of plastic inside the collector. It can also show the state of the battery if the device is powered on batteries and even set up some alarms for maintenance action.

In order to simplify the development and the deployment of the information system, all the services have been containerized with Docker^12^ and Docker-Compose^13^. To make the information system accessible to the connected devices, it requires a server with public access. An alternative to a publicly accessible server is to install Docker on a computer and use the Ngrok^14^ service, which creates a tunnel between the services on the computer and the internet. This tool is generally used for the development process and experimentation. For this article, we will consider the second scenario with the entire stack of services.

Here are the steps to follow to start the information system:

1. Assuming Docker and Docker-compose are installed on the experimentation computer, the *Information System* has to be downloaded from OSF or Github.
2. Then go to the main folder that contains the *docker-compose.yml* and *docker-compose.ngrok.yml*.
3. Some configuration has to be done before starting anything. The first thing is to create an account on Ngrok and get your Authtoken. Once you have it, you have to go to the *ngrok* folder, duplicate the *ngrok.template.yml* and rename it *ngrok.yml*. Then edit this file and modify the authtoken field by replacing *${NGROK*_*AUTH*_*TOKEN*} with the token you generated on the Ngrok website.
4. The second configuration is to duplicate the *default.grafana.env* and *default.influxdb.env* files that are in the main folder and rename them *.grafana.env* and *.influxdb.env* respectively. These two files will contain the login, the password, and other parameters that will be used to generate the database and the dashboard service.
5. Finally, the server can be started using the terminal available in VS code using this command: *docker-compose -f docker-compose.ngrok.yml up -d*. If the operation is done in a computer with the Windows operating system, make sure Docker Engine is started, otherwise the command will fail.

### 6.2 Hardware

The next stage of the building process concerns the hardware that forms the smart collector. Here is a suggested procedure to put together the different parts listed in the bill of material.

**Fig. 5:**
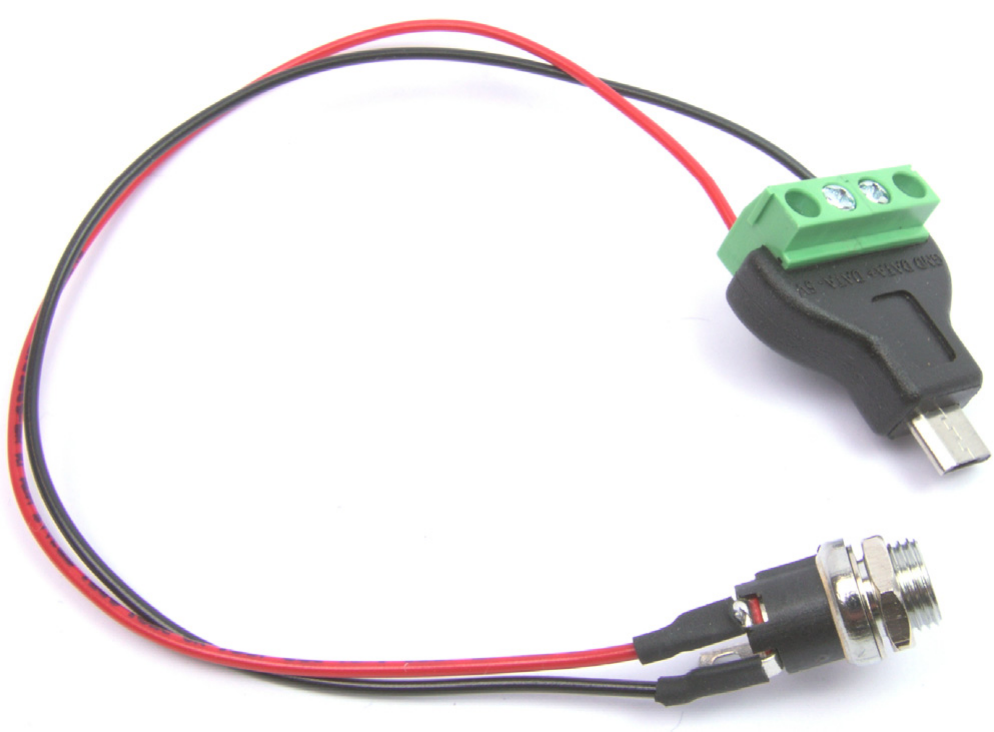
Power supply wires for the microcontroller.
  (c) Weld two wires on the button (*BTN*). To prevent shorts, you can put some heat shrink tubing on the two welds.
  (d) To secure the connection between the development board (*MCU-DEVB*) and the other electronics parts, we suggest creating a shield using a test plate (*TST-PLT*) and long connectors (*SHLD-CNT*). This shield connects the push button (*BTN*) used for taring the load cell and the amplifier circuit (*LOAD-AMP*), as illustrated in Fig. 6, to the Pycom development board (*MCU-DEVB*). To reduce the cost of the device and facilitate assembly, a PCB has also been designed to create a board that would replace the development board (*MCU-DEVB*). CAD files are available on OSF.

1. Assemble the electronic parts
  (a) Weld two wires (ideally red and black) to the power supply connector (*PWR-CNT*). To prevent shorts, you can put some heat shrink tubing on the two welds. Make sure that you solder the red and black wires to the correct pin on (*PWR-CNT*).
  (b) Connect two cables to the USB adaptor (*USB-ADPT*), taking care to connect the red wire to the terminal block that corresponds to the USB positive pin and to the black wire respectively. The results may look like Fig. 5.

**Fig. 6:**
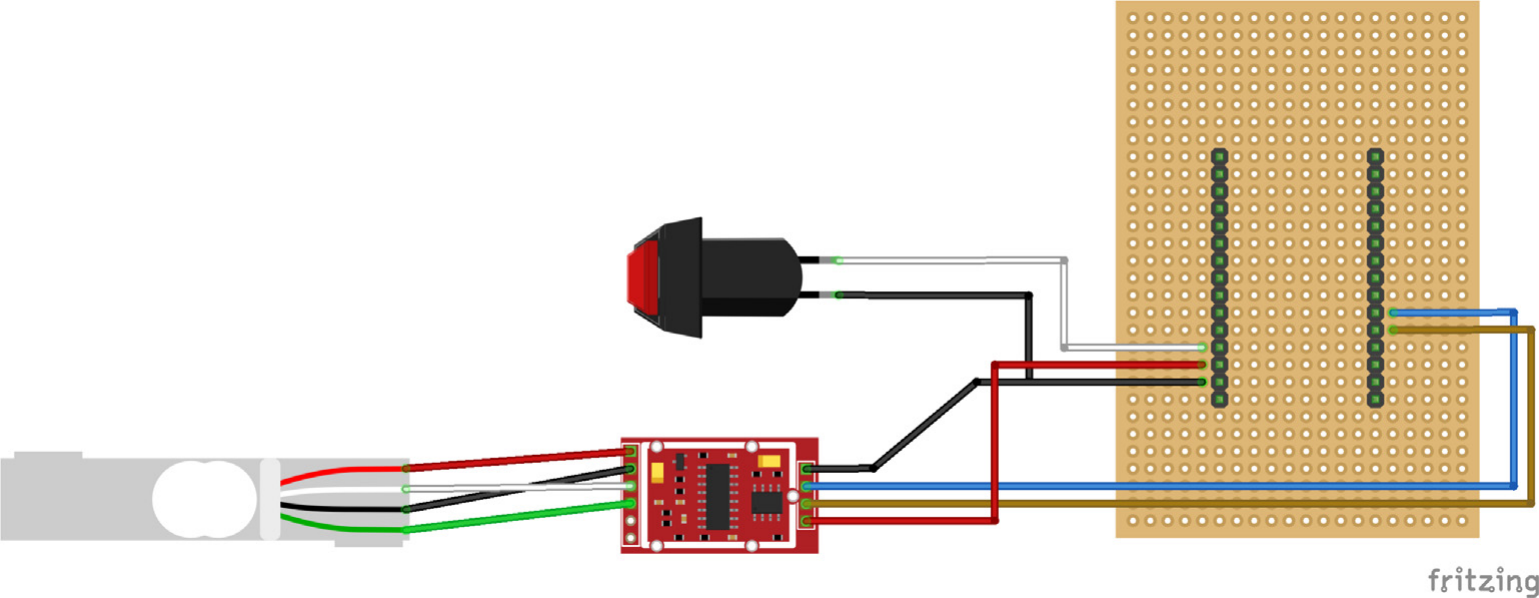
Shield soldering schema.
2. Make two H-shaped assemblies with three aluminum profiles (*PRF2020*) and four brackets (*BRK*), respectively. The first assembly will have the middle profile at 70.5 mm from the front (Part A) and the second at 69.5 mm from the front (Part B). To simplify the assembly, all the aluminum profiles have the same 200 mm length. Fig. 7 provides more insight into how the assemblies look.

**Fig. 7:**
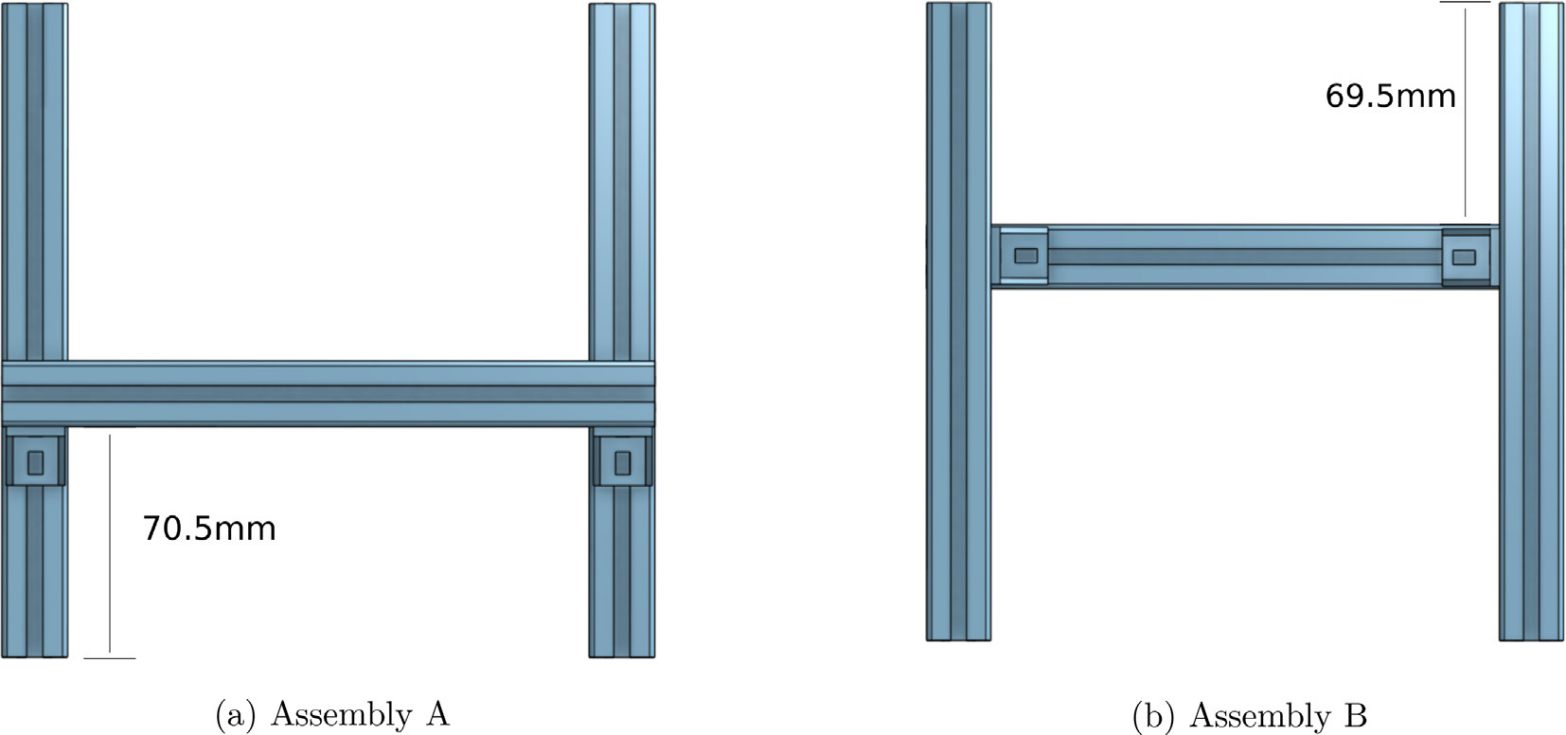
Two assemblies of aluminium profile for the base.
3. Put together plates that will maintain and protect electronic parts.
  (a) Assemble the *base bottom* plate with assembly A using four M3 screws and T-shaped nuts.
  (b) Assemble the *base back* plate with assembly A using two M3 screws and T-shaped nuts.
  (c) Join together the button (*BTN*), the power supply connector (*PWR-CNT*), and the external antenna connector (*EXT-ANT*) on the *Base Front* plate as illustrated in Fig. 8. Then the plate is screwed to assembly A using two M5 screws. As the hole of the aluminum profile is not threaded, it is necessary to apply a torque to create the thread while it is screwed.

**Fig. 8:**
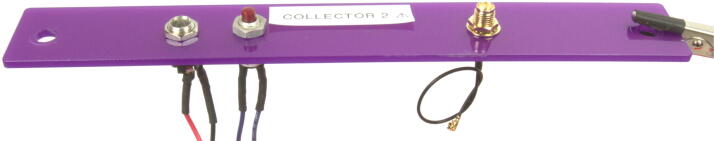
Connector and tare button on the Base Front plate.
  (d) Screw the development board (*MCU-DEVB*) and the amplifier HX711 (*LOAD-AMP*) to the bottom plate with M3 bolts and using the mounting plate (*Base Fix*) to crimp the amplifier board (Fig. 9).

**Fig. 9:**
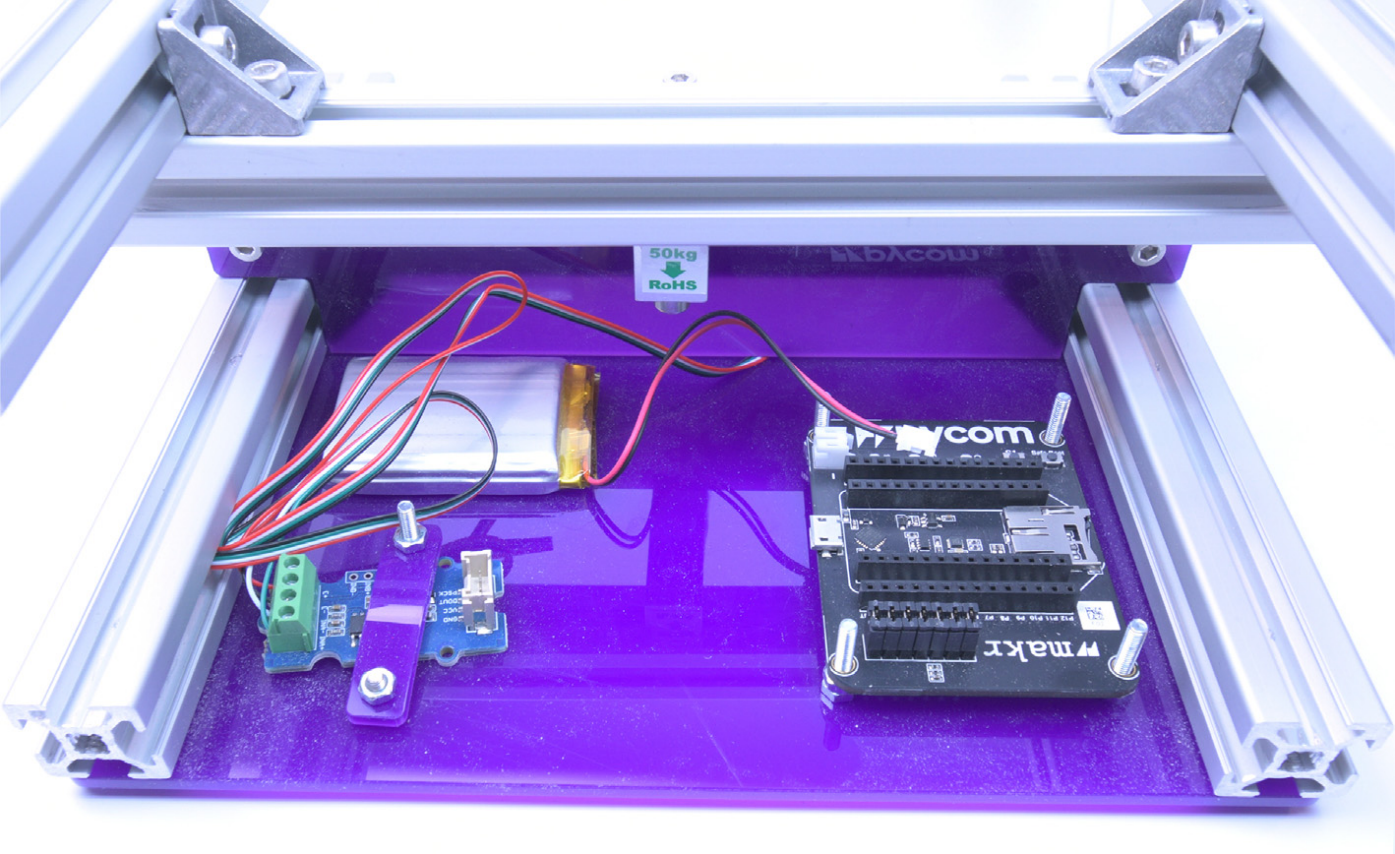
Mounting electronics in the base.
  (e) Then put the shield illustrated by 6 on the Pycom development board (MCU-DEVB). When everything is connected, it should look like 10.

**Fig. 10:**
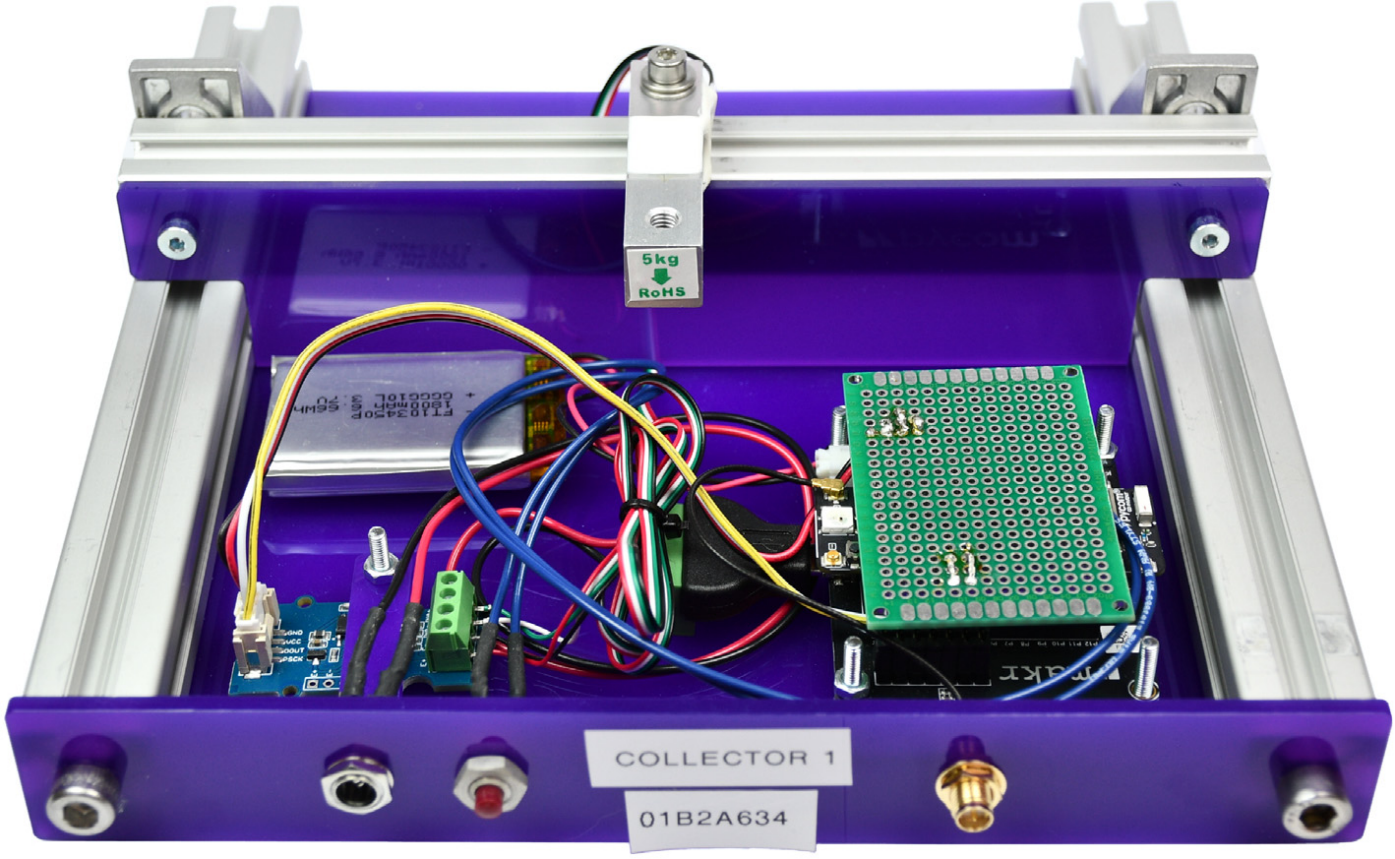
Connector and tare button on the Base Front plate.
  (f) Finally, close the case with the spacers (*Base Spacer*), the top plate (*Base Top*), and M3 screws and T-nuts.
4. Put together plates that will form the collector.
  (a) Assemble *collector front*, *collector back*, and *collector bottom* using the four *collector fasteners*, two on each side as shown in Fig. 11.

**Fig. 11:**
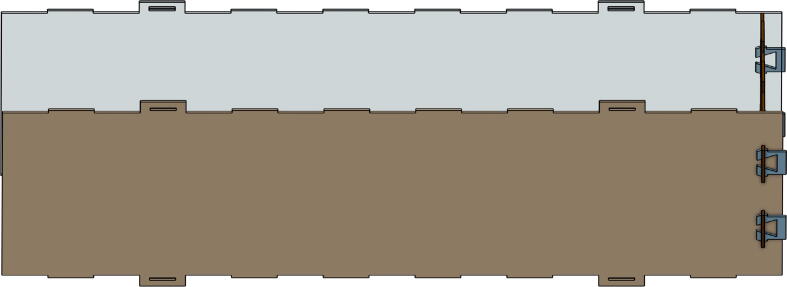
Assembly of the front and back plates to the bottom of the collector.
  (b) Put the *collector right* and *collector left* plates to the previous assembly using eight *collector fasteners* as shown in Fig. 12.

**Fig. 12:**
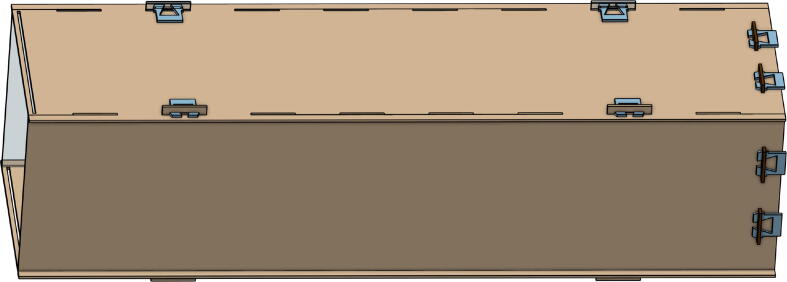
Assembly of the left and right plates to other parts.
  (c) Slide the *collector top* to close the collector.

### 6.3 Firmware and embedded program

There are two distinct aspects related to programming the microcontroller of the smart collector:

• The firmware that handles the Micropython runtime, the bootloader and default libraries specific to Pycom microcontroller.
• The embedded program that is written in Micropython and that will do the measuring and communicating with the information system.

**Fig. 13:**
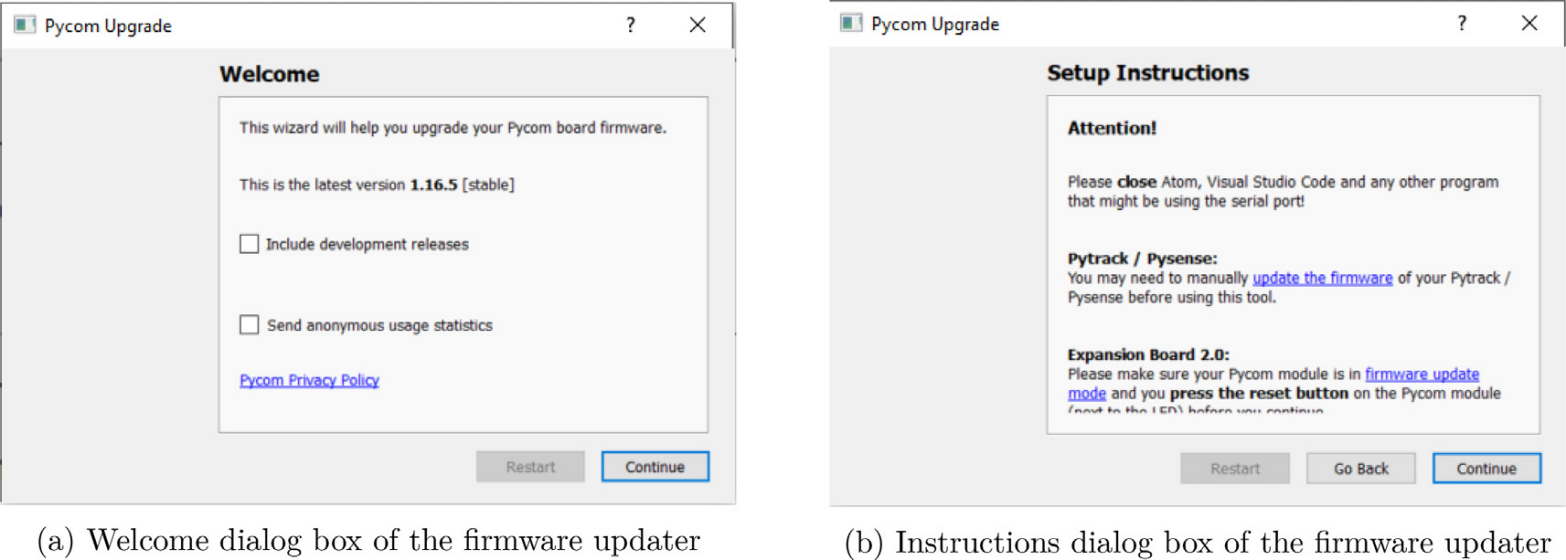
Firmware updater dialog box.
  (b) The program gives you some instructions depending on the MCU or the expansion board you are using. A frequent source of issue is to keep VS code open when updating the firmware, so take care to close it before starting the upgrade (Fig. 13b). If it is OK, click on continue.
  (c) Define the communication setting to do the update. You have to identify the port where the MCU is connected. The default speed is set to 115200 baud. Take care to check the “Show Advanced Settings” (Fig. 14a).

The firmware is provided by Pycom and may require some updates according to the improvement and bug fix. The embedded program is the program that has been written by the author to collect data and send it to the information through Sigfox. To program the microcontroller, this would require three different software items:

• The Visual Studio code IDE
• The Pymakr^15^ plugin for Visual Studio Code
• The Pycom Firmware updater^16^

1. Update the firmware of the microcontroller.
  Before uploading the program on the microcontroller, Pycom recommends updating the firmware. This update requires the Pycom Firmware updater.
  (a) Start the program; it will prompt a welcome dialog box (Fig. 13a). You must click on continue.

**Fig. 14:**
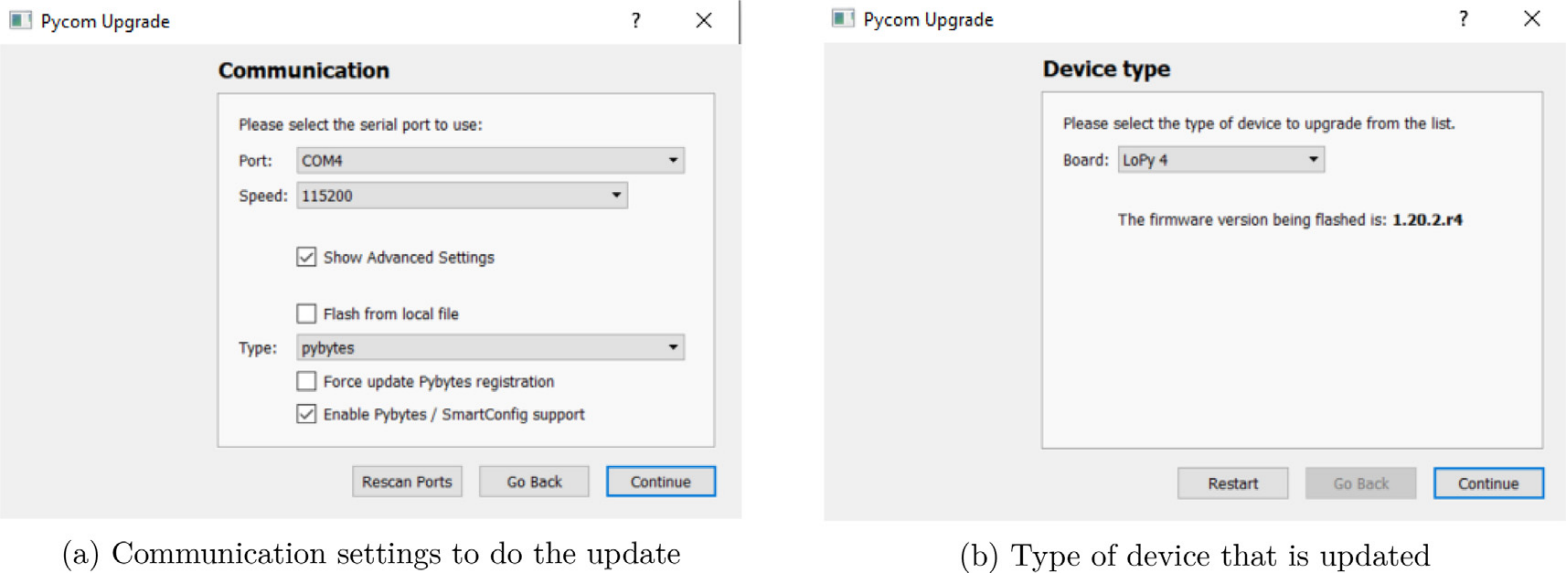
Setting dialog box of the firmware updater.
  (d) In order to flash the proper firmware, it asks to validate the board used. In this work, it is LoPy4 (Fig. 14b).
  (e) As the standards concerning communication frequencies are not the same according to the localization, the firmware updater asks the region for both the LoRa and Sigfox connectivity. In this project, it was Europe and France (Fig. 15).

**Fig. 15:**
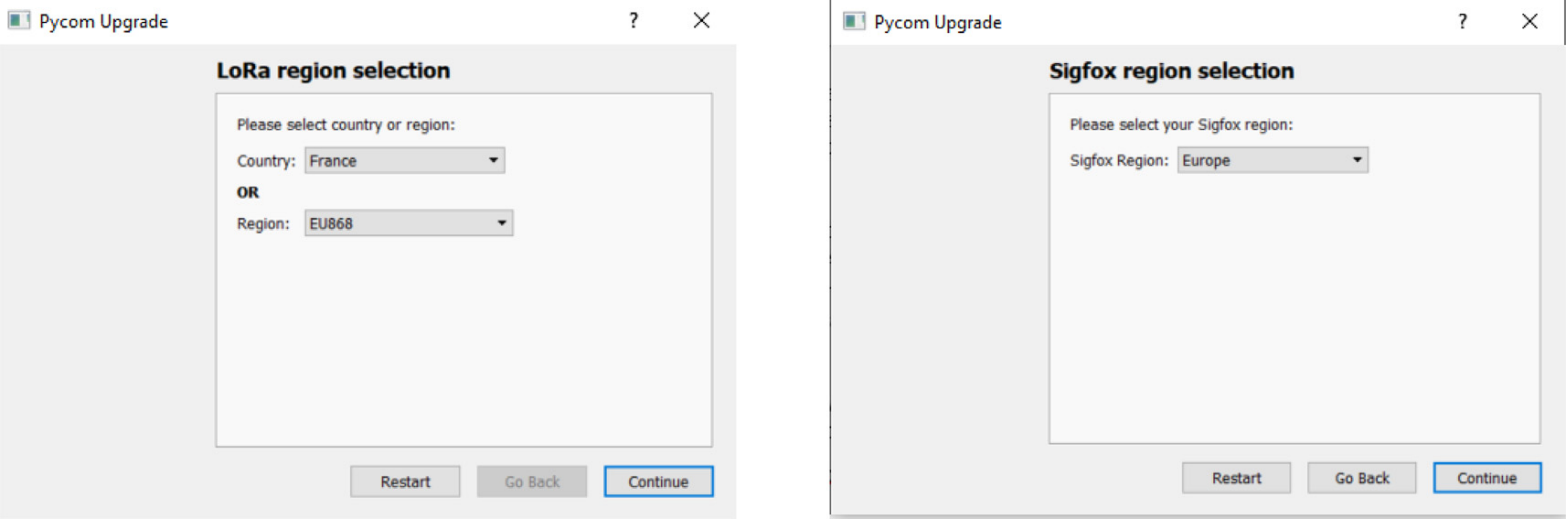
Region selection dialog box of the firmware updater.
  (f) As the “Show Advanced Setting” box was checked (Fig. 14a), the firmware updater will sum up the previous settings and the formatting parameters such as the system file (Fig. 16a).

**Fig. 16:**
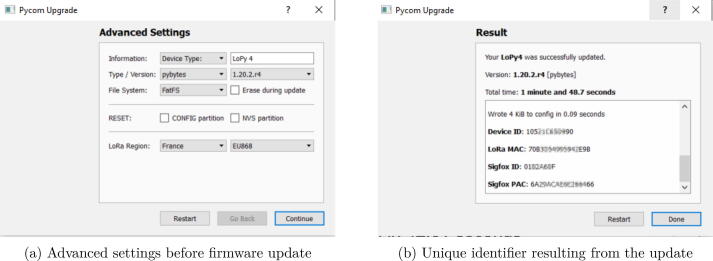
Firmware update setting and results.
  (g) Finally, after waiting several minutes, a dialog box will prompt you about whether the update was successful or not (Fig. 16b). The windows will also contain information concerning the device and notably the Sigfox ID and the Sigfox PAC that will be used in the next step. For commodities, this information can be saved in a spreadsheet available on OSF ( https://osf.io/95a6w/).
2. Register the microcontroller on Sigfox Backend.
  In order to use the Sigfox network, you must register your device. The Pycom documentation provides the elements of the procedure to register the device^17^. In short, you must go to the Sigfox activation page^18^ and create an account. If it is not your first device, make sure you sign in on the platform. The first step asks for your deployment country, then you must enter the Sigfox ID in the Device ID form and enter the PAC number (Fig. 17). Then, depending on whether or not you already have an account, it asks you to create one before validating the registration of the device.

**Fig. 17:**
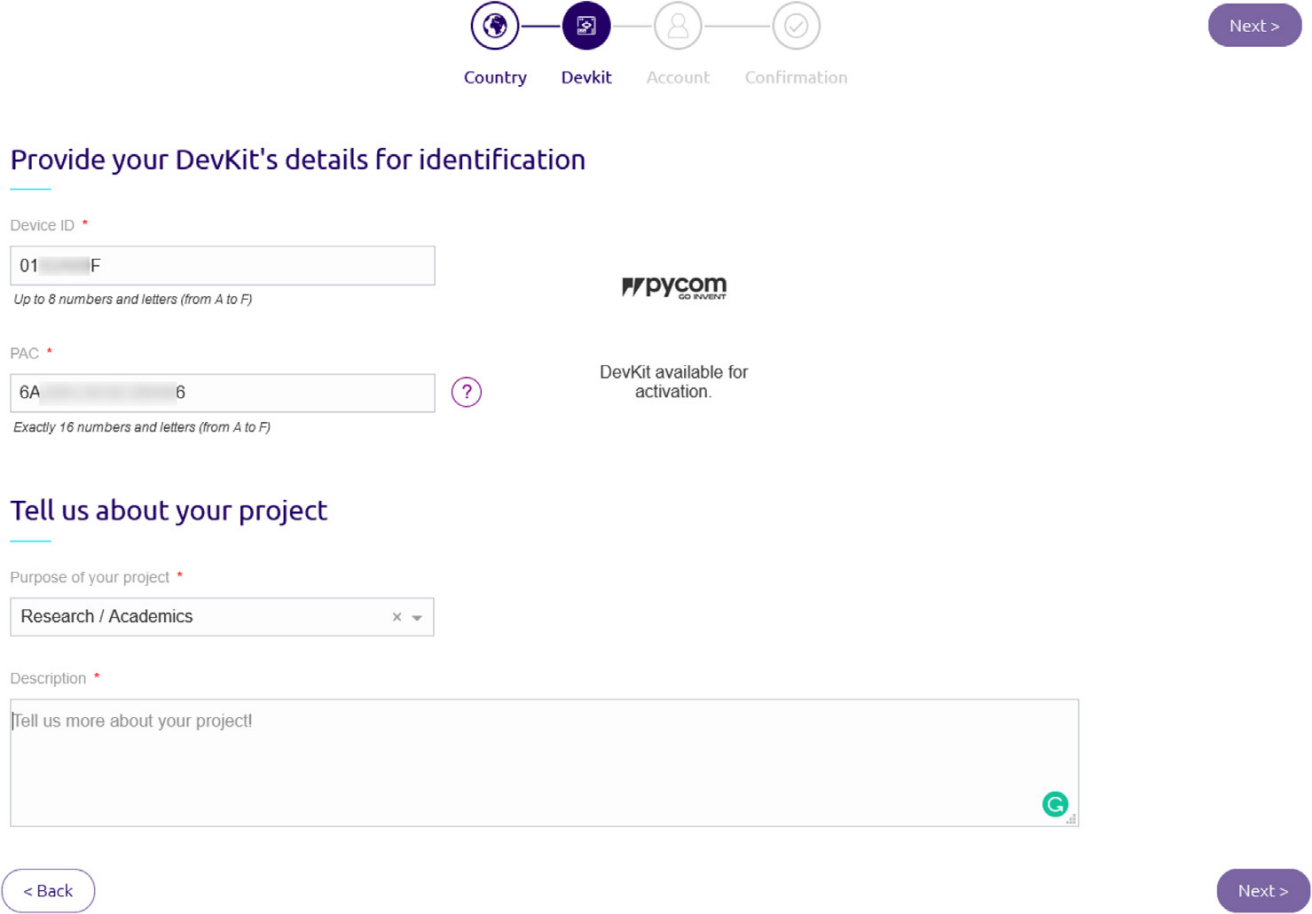
Sigfox activation form for adding a new device.
3. Calibrate the load cell.
  By default, connecting the load cell to the microcontroller will not provide the correct weight. A calibration procedure is necessary to determine the calibration factor. This would require calibration weights. An alternative, which has been used for this project, is to replace calibration weights with weightlifting weights. The drawback is the imprecision of the indicated weight. To mitigate this, each weightlifting weight was weighed ten times using a laboratory scale and then the readings were averaged. To simplify the later calibration tasks, the authors suggest putting a tag or anything that enables the identification of the weight and its real weight. Once the true value is known for each weight, the calibration procedure can begin.
  (a) Connect the smart collector to a computer through the development board (MCU-DEVB) with a USB cable.
  (b) Start Visual Studio Code, open the firmware folder downloaded from OSF ( https://osf.io/wrzpe/) and start a connection with the microcontroller using Pymakr. To do so, click on the Pymakr Console button at the bottom of the Visual Studio Code window (Fig. 18).

**Fig. 18:**
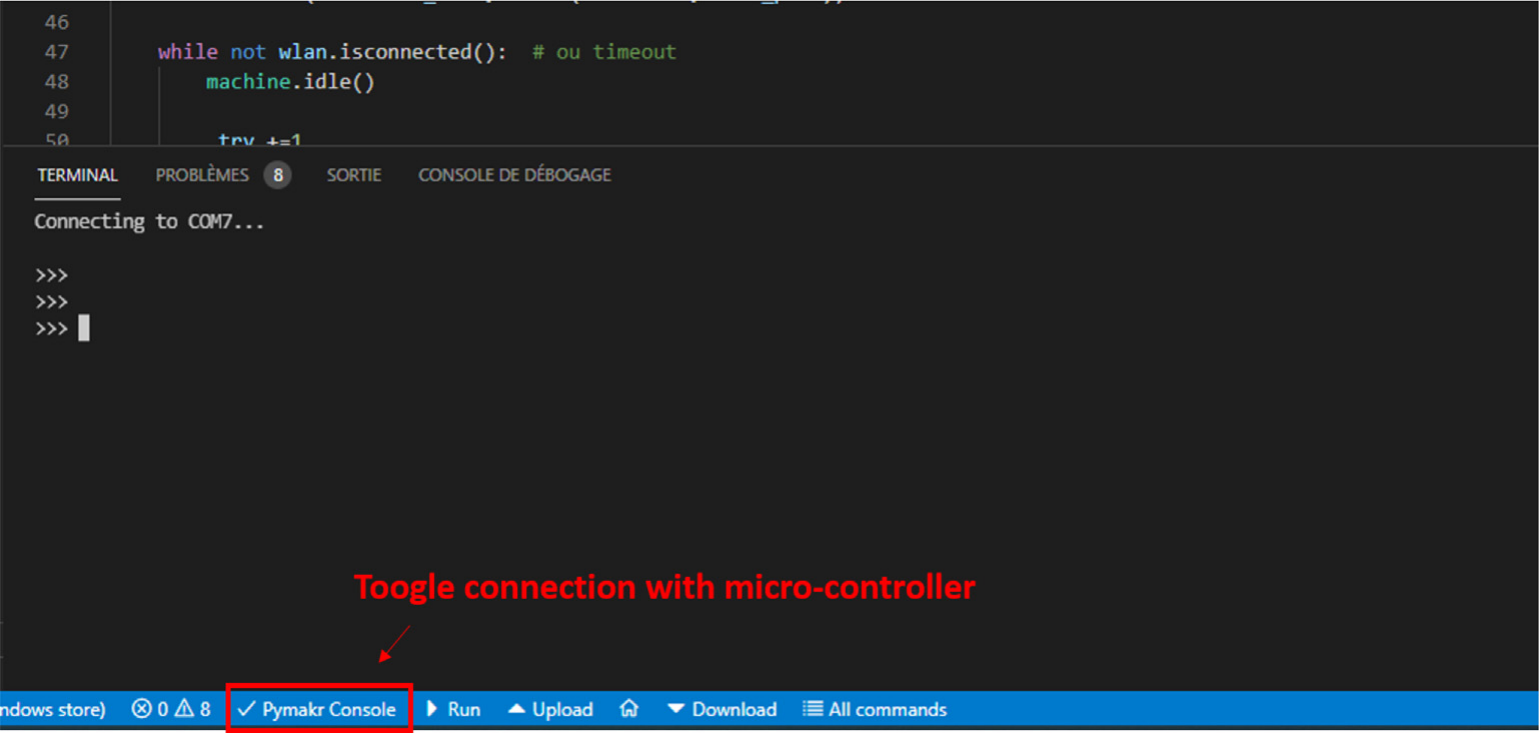
Activate the connection with the microcontroller.
  (c) Start the program called *callibration.py* using the command ”*callibration.py*”. It will prompt instructions in the console and notably specify to the user which keyboard keys to hit to increase or decrease the calibration factor. The principle of this program is to iteratively change the calibration factor (also called scale in the HX711 library) to measure the closest weight of the calibration weight.
  (d) Put one or several weights on the load cell, take a measurement and adjust the calibration factor to reach the closest value of the expected weight.
  (e) Once satisfied with the measurement, keep the real weight on the load cell, the measured weight and the calibration factor in a spreadsheet. The configuration spreadsheet available on OSF ( https://osf.io/95a6w) has tabs for each load cell that illustrate this.
  (f) Repeat the previous step with additional weight while being careful not to exceed the maximum weight.
  (g) After reaching the maximum weight, average the calibration factor collected previously to determine the calibration factor of the load cell. Based on the experience of the authors, it is suggested to label the load cells and maintain a file with microcontroller information (Microcontroller type, Device ID, LoRa MAC, Sigfox ID, Sigfox PAC, state), load cell information (ID, max weight, calibration factor) and to describe the composition of each smart collector. An example of the spreadsheet is available on OSF ( https://osf.io/95a6w/).
4. Configure the program and upload it.
  Up to now, the microcontroller has had the lastest firmware and is registered on Sigfox Backend to enable LPWAN connectivity. However, it does not have the expected functionality for the smart collector, which means doing a periodic weight measurement and sending data via LPWAN. To do so, here are the steps to follow:
  (a) Start Visual Studio Code, open the firmware folder downloaded from OSF ( https://osf.io/wrzpe/) and start a connection with the microcontroller using Pymakr.
  (b) Modify the “*Main.py*” file to enter the credentials of the Node-RED API as well as the microcontroller parameters such as the calibration factor of the load cell or the time between two messages (Fig. 19).

**Fig. 19:**
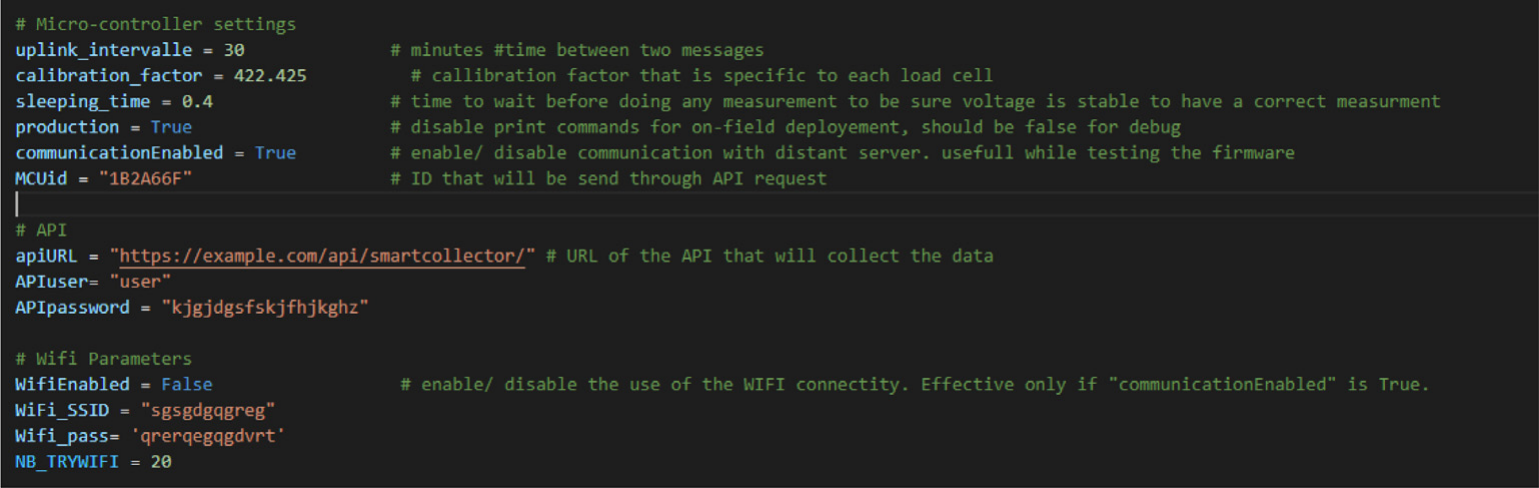
Parameters in the firmware.
  (c) As for the firmware update, put the LoPy4 (MCU) on the development board (MCU-DEVB). Take care to properly connect the antenna (EXT-ANT) to the microcontroller (MCU) because using the Sigfox feature without the antenna may damage the microcontroller. Finally, connect the dev-board (MCU-DEVB) to the computer.
  (d) Identify the port which is used by the computer to communicate with the MCU-DEVB. Pymakr has a functionality to list the port used as illustrated in Fig. 20. It is accessible through the “*All commands*” button then “*Pymakr* 〉 *Extra* 〉 *List Serial Ports*” item in the drop-down list.

**Fig. 20:**
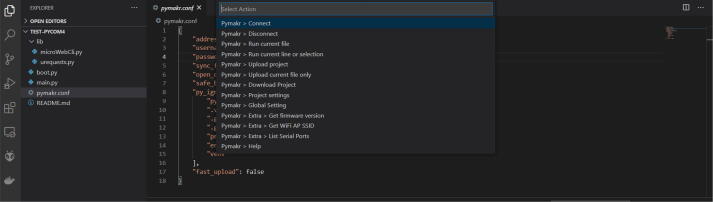
List the port used by the computer.
  (e) Modify the *pymakr.conf* configuration file at the line *address* to define which port to use to upload the program (Fig. 21). The port changes according to the *MCU-DEVB* used, so if you have several dev-boards, do steps 4 and 5 each time you change it.

**Fig. 21:**
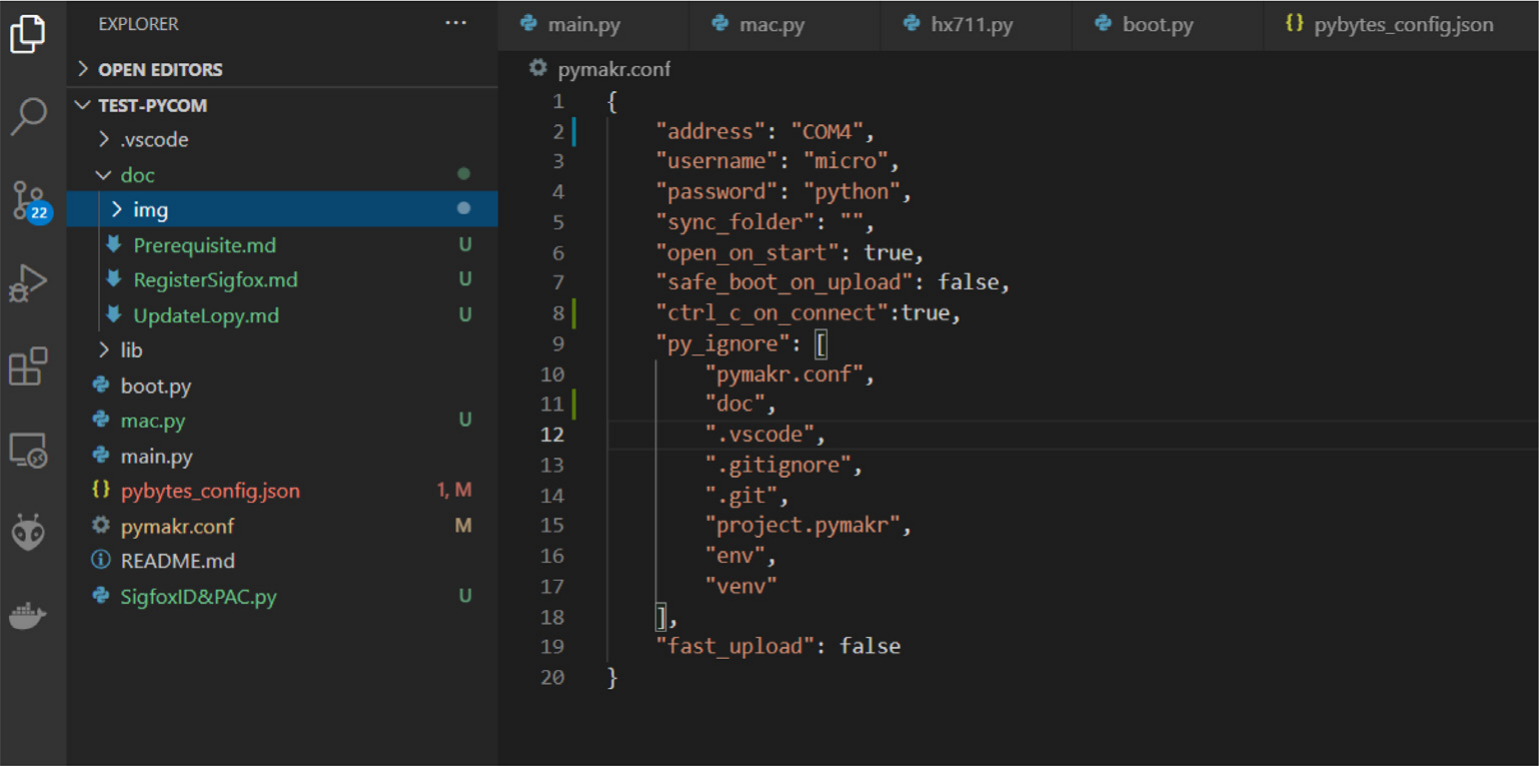
Modification of the configuration to allow the connection between the computer and *MCU*.
  (f) Establish a connection between the *MCU* and the computer. Click on the button ”Pymakr Console” (Fig. 18); this will make the console appear, together with the logs. Then click on ”*Upload*” to start uploading the program into the *MCU* (Fig. 22). The progress will be prompted in the terminal.

**Fig. 22:**
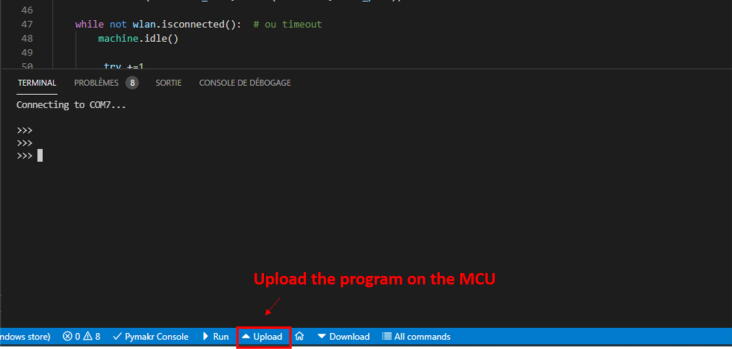
Upload the program on the *MCU*.

## 7 Operating instructions

**Fig. 23:**
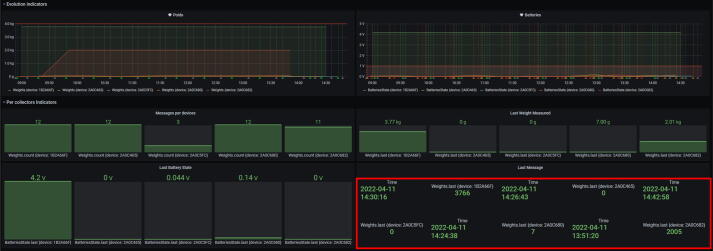
Last message date and value of each smart collector on the dashboard.

Once the Smart Collector is assembled, configured, and connected to the information system, operation is quite simple as there is only one button. If the collector is powered, it has two states: awake and asleep. The system spends most of the time asleep to save energy. This is essential when it is not connected to a power outlet using the 5 V power supply (*PWR-SP*). When awake and tared, the system measures the weight, sends it to the Sigfox back-end and then falls asleep. The system will wake up several seconds every 30 min according to the default settings in the program. When the smart collector is not tared, the device will wake up, check its memory, and fall asleep. To tare it, you have to push the only button available on the collector. It will wake up the microcontroller to weigh the collector preferably without any plastic inside, save it to the persistent memory, and send a message to the back-end. During the awake phase, the built-in LED of the microcontroller allows the user to know the state of the device according to color codes defined by Table 4. While asleep, there is no way to see directly on the collector whether it is operational. To do so, users have to check the online dashboard to see the last communication as shown in Fig. 23.

**Table 4:** Color coding of the microcontroller when taring or communicating

**Color**	**Code hexa**	**Meaning**
	#007F00	Measuring the weight of the collector
	#7F0000	Tare data not found in the persistent memory
	#007F7F	Wake up from reset button
	#00007F	Wake up from tare button
	#FFF81B	Wake up from RTC time out

Then, in addition to the information system infrastructure, an organization has to be defined in order to retrieve material that has been collected or load the collector if it runs on batteries. The dashboard can further assist this step through alarms when a weight threshold has been reached by a collector. According to the capacity test, a weight threshold has been set to 4 kg of plastic bottle caps. Another alarm was set up for the battery’s state of charge to know when to charge the collector. These alarms can even be connected to communication channels such as email or Telegram.

## 8 Validation and characterization

**Fig. 24:**
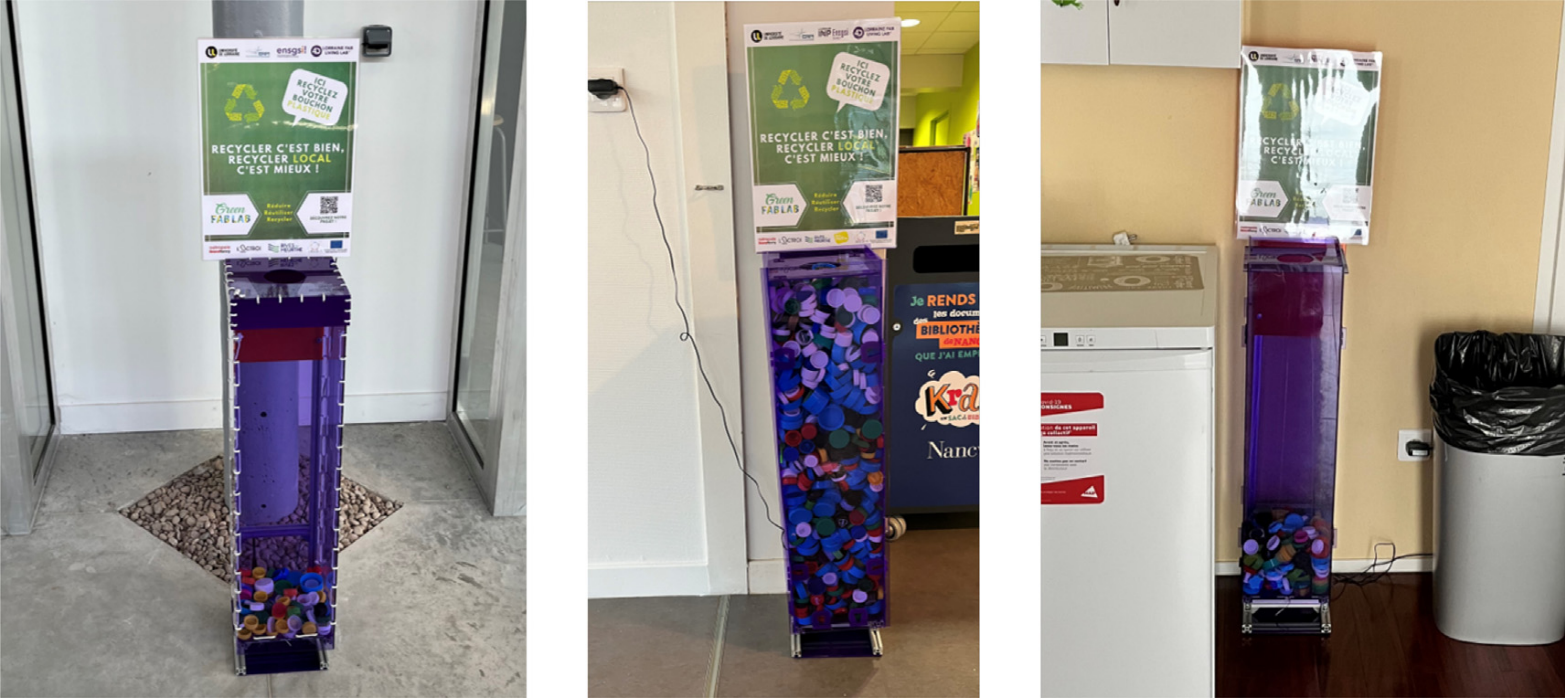
Examples of Smart Collectors on site.

**Fig. 25:**
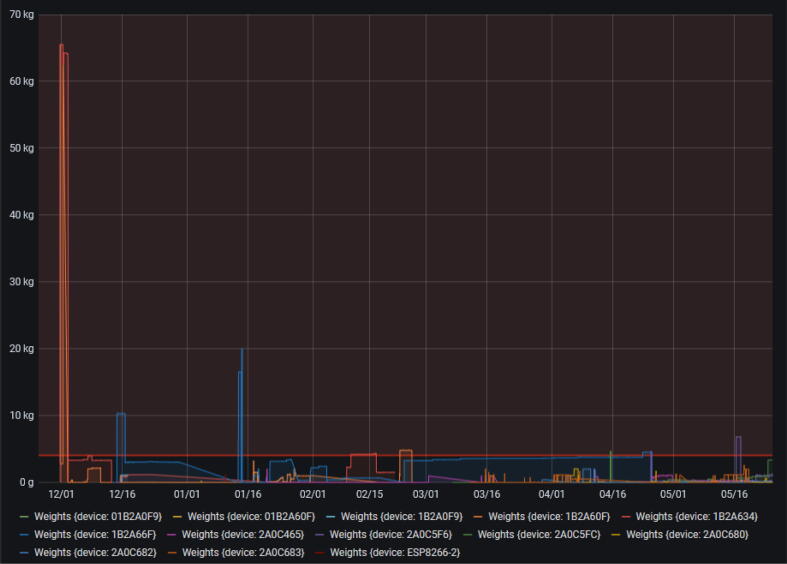
Weight measurements over time from the various prototypes created.

The smart collector was deployed in the field to collect plastic waste for the purpose of recycling it locally. Fig. 24 illustrates several implementations of smart collectors in the district of Rives de Meurthe in Nancy, France. The collected plastic was transformed into usable recycled injected components (sheets, recycled rods) using open hardware as extrusion machines and hydraulic press machines. The main purpose was also to obtain 3D printing filaments or usable shredded pellets for fused granular fabrication [27]. The minimal viable collection process was limited to bottle caps, given the level of contamination taken into account for facilitating the recycling process. This first definition of the scope was based on the relative homogeneity of the possible waste. However, different type of plastics are used on the market for plastic objects. In the long run, the data gap that the smart collector aims to fill is to better understand the production of plastic waste in the function of type of entity, e.g. public, private or social. Also, the creation of granular data on a neighborhood scale, enables us to better dimension what could be a closed-loop recycling process at this scale. From this perspective, the creation of eight collectors generated 27,678 rows of value from September 22, 2021 up to May 25, 2022 (Fig. 25). This data will be used to improve the local recycling supply chain [28], [16]. However, it cannot be used straight away as it has to be cleaned before use.

As shown in Fig. 25, there are outliers due to a bug in the former version of the collector and technical issues in the field. The program can be considered as behaving correctly since February 2022, so outliers are mainly due to issues in the field, such as people putting object on the collector. Even if it is only five minutes for a chat during a coffee break, this is enough to produce an outlier if the MCU schedule is reached.

Due to the difficulty in obtaining electronic components, we have had to deal with different microcontrollers for prototyping smart collectors. Some have both LoRa and Sigfox (LoPy4^19^ microcontroller) connectivity, whereas others have only Sigfox connectivity (SiPy^20^ microcontroller). The original design included a LiPo battery in order to position the collector wherever the user wants to increase the collection. However, in practice, the autonomy was confusing in comparison to the common ESP32^21^ microcontroller. Based on the power-consumption specification from the respective data sheets summarized in Table 5, it is possible to estimate their lifespan based on a 3.7 V LiPo battery with 2000 mAh of capacity. To do so, the same cycle of communication was considered for the three microcontrollers and was composed of three steps which correspond to the different states of consumption:

• Deep sleep when the device is waiting.
• Idle when the device wake up and is doing calculation without communication
• Communication which uses either Sigfox of WiFi according to the microcontroller

**Fig. 27:**
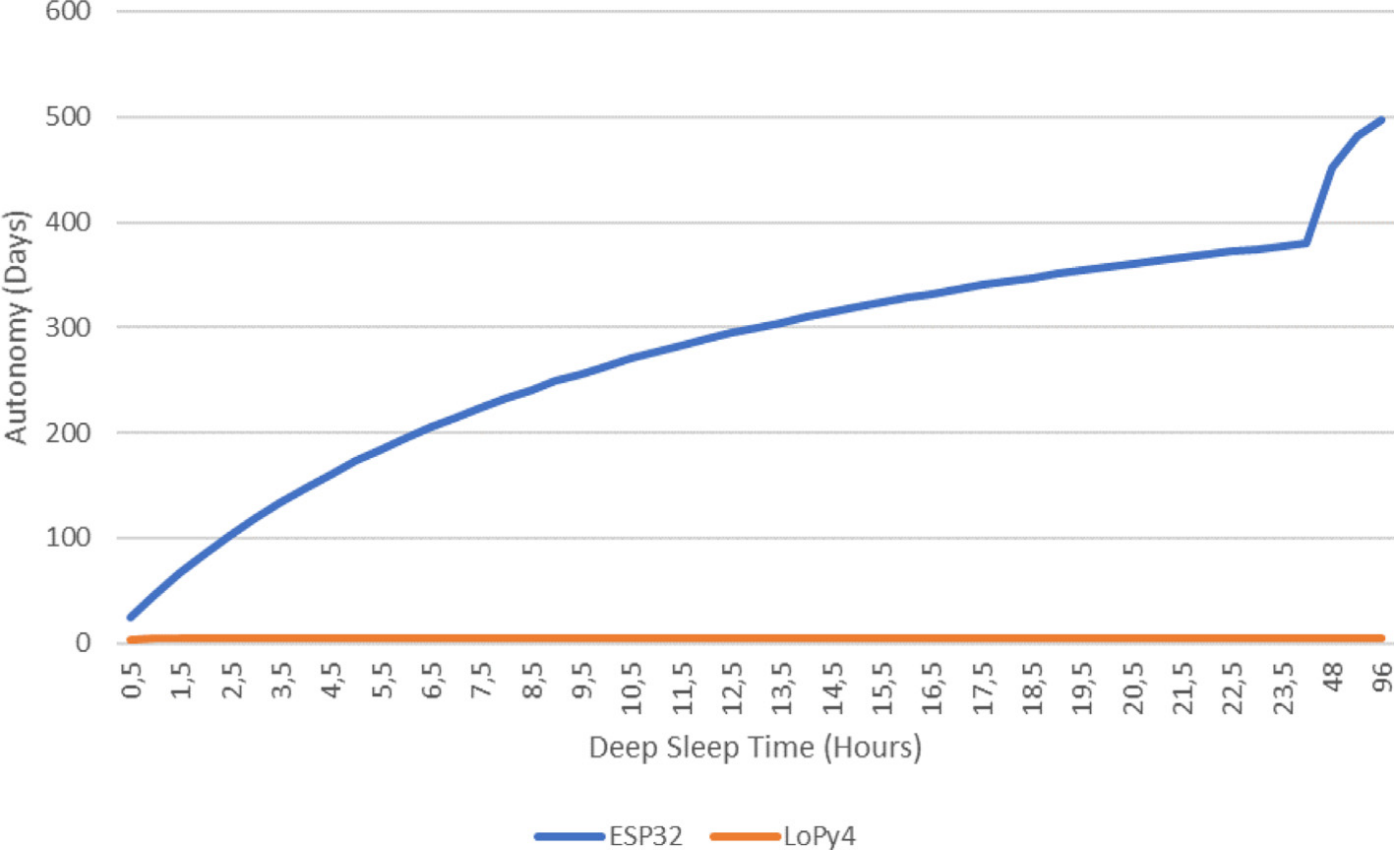
Comparison of the deep sleep time on the autonomy for ESP32 and LoPy4.

Based on these steps, the time of ther idle and communication steps was experimentally determined, as illustrated in the Table 6. This data allows for the calculation of the consumption per cycle with the Eq. 1 where $E_{\mathrm{cycle}}$ is the consumption per cycle, $I_{\mathrm{com}},I_{\mathrm{idle}}$, and $I_{\mathrm{ds}}$ are the current drawn during communication, idle and deep sleep steps respectively. Similarly, $t_{\mathrm{com}},t_{\mathrm{idle}}$ and $t_{\mathrm{ds}}$ are the time spent on each step. Then the autonomy is calculated with the Eq. 2 where $t_{\mathrm{cycle}}$ is the time of the cycle, which sums $t_{\mathrm{com}},t_{\mathrm{idle}}$, and $t_{\mathrm{ds}}$. $E_{\mathrm{bat}}$ and $E_{\mathrm{cycle}}$ are the power stored in the battery and the power consumed during a cycle, respectively. These two values have to be given in the same unit of measurement, meaning either mAh or Ah. The denominator of the equation is the number of seconds in one day, used to give autonomy in days. The results are summarized by the autonomy column in Table 7. It shows that microcontrollers which embed the Sigfox connectivity, known as low-power wide-area network connectivity, ultimately have less autonomy than the ESP32 microcontroller that uses WiFi. This is not a connectivity issue; the power consumption of the LoPy4 while communicating with Sigfox is lower than the ESP32 communicating with WiFi. The issue comes from the power consumption of the Pycom microcontrollers when they are in deep sleep mode. A solution could be to send less frequent messages and to spend more time in deep sleep mode to save power. A quick extrapolation of the time in ”deep sleep” mode leads to Fig. 27, which shows a real increase in the autonomy for the ESP32. On the other hand, the LoPy4 MCU has no impact on autonomy; it remains around 4.2 days. These calculations remain theoretical; in practice, smart collectors based on LoPy4 have autonomy higher than four days. However, the order of magnitude remains the same; smart collectors based on ESP32 have better autonomy. Given that short autonomy implies logistics and handling, the decision was made to keep SiPy and LoPy4 MCU and power it through a power socket adaptor. This is less convenient since you have to install the collector near an electrical outlet but it simplifies the network connectivity.

(1) $$E_{\mathrm{cycle}}=I_{\mathrm{com}}\times t_{\mathrm{com}}+I_{\mathrm{idle}}\times t_{\mathrm{idle}}+I_{\mathrm{ds}}\times t_{\mathrm{ds}}$$

(2) $$T_{\mathrm{bat}}=\frac{t_{\mathrm{cycle}}\times E_{\mathrm{bat}}/E_{\mathrm{cycle}}}{86400}$$

**Table 5:** Consumption of the microcontrollers according to their mode.

Mode	Consumption (mA)		
	LoPy4	SiPy	ESP32
idle	40	37.7	100
Sigfox TX RCZ1	42	47	N/A
WiFi	107	111	180
Deep Sleep	19.5	15.1	0.15

**Table 6:** Consumption of the microcontrollers used according to their mode.

MCU	Communication		Idle		Deep sleep	
	Current(A)	Time(sec)	Current (A)	Time(sec)	Current (A)	Time(sec)
LoPy4	0.042	3	0.03	40	0.0195	1800
SiPy	0.042	3	0.0377	40	0.0151	1800
ESP32	0.18	25	0.1	15	0.00015	1800

**Table 7:** Consumption and autonomy of each microcontroller with a 2000 mAh LiPo battery.

MCU	Consumption per cycle (Ah)	Cycle duration (sec)	Cycle number	Autonomy (days)						
LoPy4	0.0101	1843	197	4.2						
SiPy	0.008	1843	249	5.3						
ESP32	0.0017	1845	1148	24.5

## 9 Conclusion and perspective

This paper presents the technical development of a smart plastic waste collector that is used in the context of an experiment for a distributed recycling approach. The main scientific interest in the development of this collector is to contribute to the smart waste management [6], [29] field, with the long-term aim to help in the operational design of a closed-loop waste supply chain for plastic waste for distributed recycling [30], [16], [21]. The technical reasons to develop this device rely on the possible identification of useful plastic waste that can be recycled. It is essential for the creation of an open-source hardware ecosystem to recycle plastic, including extrusion, such as the RepRapable Recyclebot [31]. The collection process is an essential step in mechanical plastic recycling. Nevertheless, it is also necessary to consider sorting, shredding, cleaning, and compounding processes [21] for future open hardware development, as maker initiatives such as Precious Plastic^22^ are seeking.

Over the nine months of deployment and experimentation (from January 2022 to September 2022), about 60 kg of plastic were collected. The collection process was limited to bottle caps because of the low level of contamination, thereby facilitating the recycling process. This first scope enables the relative homogeneity of the possible waste. The collection was carried out in an urban area with a radius of 1 km. For this reason, the technical solution was oriented toward the use of a Wide Area Network such as LoRaWAN or Sigfox. As the ambition was initially to avoid the complication associated with managing network infrastructure such as a LoRa gateway, it was decided to use Sigfox. This decision reduced the number of compatible microcontrollers to two main solutions: Pycom LoPy4 and Aruino MKR FOX 1200, one of which offers four connections. This solution, attractive at first sight, turned out to be a bad decision in regard to its battery operation. Furthermore, it increased the price of the device, which reduced its scalability. The device which was developed remains in a prototype state that would require further engineering in an electronic perspective to have more accessible and cheaper electronic components. The material was sorted by material type and by color. Based on this stock, several objects and furniture parts, such as table stands and handles 26, were able to be manufactured. The manufacturing process consisted of desktop injection molding, and a manual sheet press. Currently, this unit remains at the stage of exploration for the different ways to value the plastic in order to create plastic products for local designers inside a maker ecosystem. The results of this experiment will enable better understanding of how a distributed recycling unit can be implemented in a local territory. A parallel collection campaign is taking place to collect plastic bottles from a specific brand of water with the purpose to explore fused granular fabrication [32].

**Fig. 26:**
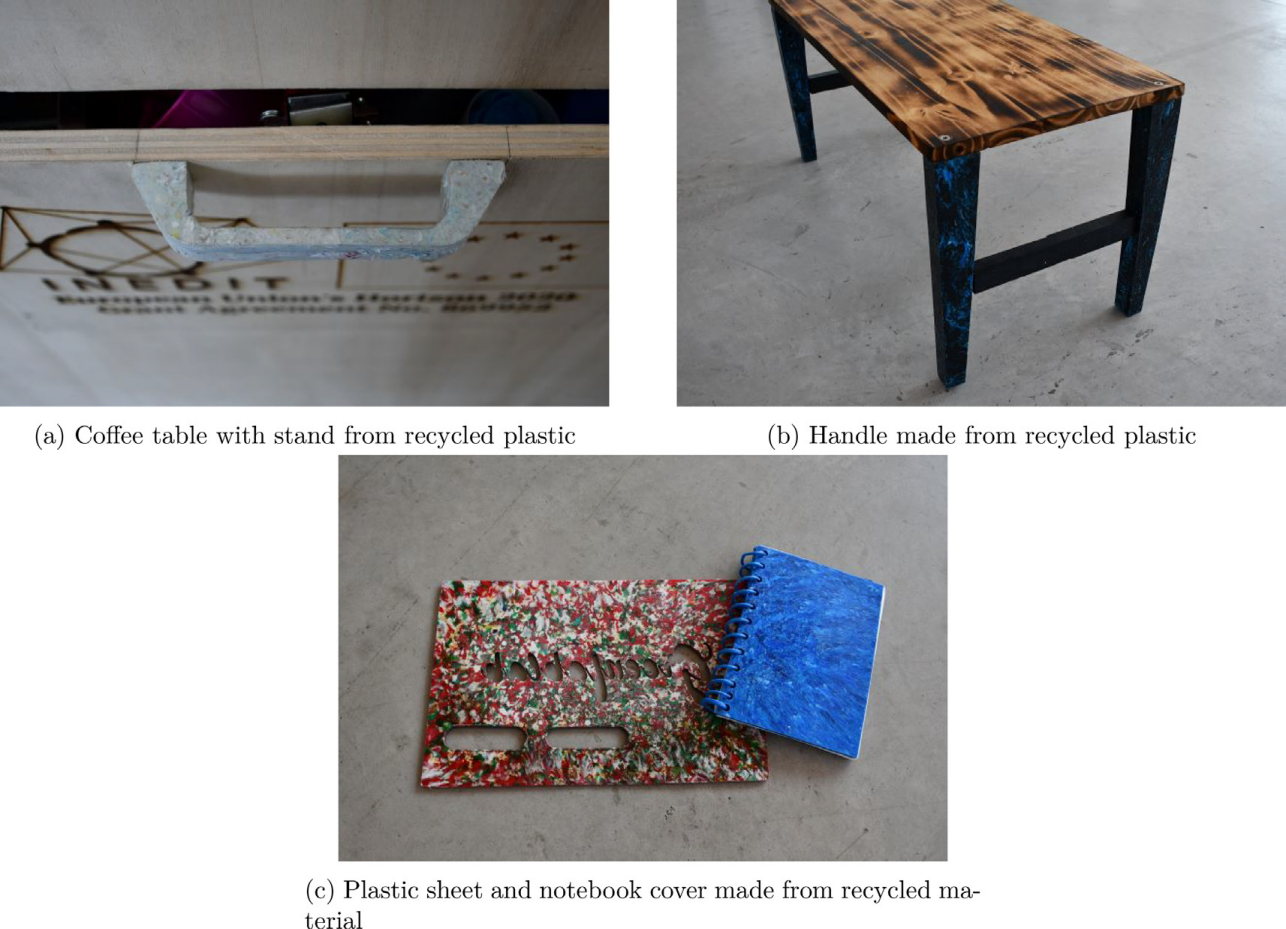
Example of production made from recycled plastic.

Based on experience in the field, several improvements for future versions of the smart collectors can be proposed, such as:

• Simplifying the calibration process. Authors are thinking of gathering the calibration program in the main program file and enabling/disabling with one variable.
• Removing the development board from the deployed prototype, as the battery is not used anymore, and replacing it with a custom shield. This will slightly reduce the cost of the prototype. If a battery remains a requirement, an alternative to the development board is a battery management system based on a TP4056 chip.
• Changing the microcontroller to something that consumes less power in order to allow more mobility for the smart collector and not depend on power outlets.
• Deploying a LoRaWAN gateway to try and compare a LoRa-based solution with the current version based on sigfox.

Except for its application in the domain of plastic collection, this device expands the capabilities of usage of a connected load cell. With the appropriate calibration, it can be transformed into connected lab-grade scales [33]. For larger weight, the principle of the device is quite similar to a smart weighing scale for beehive monitoring [34]. For even larger weight and movement analysis, more load cells might be connected to the microcontroller, which would allow gait analysis such as [35]. Another application might be the integration into a shelf tracking system [36]. In association with other sensors, connected load cells can be used to monitor anything, such as Smart Gas Management System [37] that measures the amount of gas lost through a gas leak.

## CRediT authorship contribution statement

**Alex Gabriel:** Conceptualization, Methodology, Software, Data curation, Writing - original draft. **Fabio Cruz:** Conceptualization, Methodology, Writing - review & editing.

## Declaration of Competing Interest

The authors declare that they have no known competing financial interests or personal relationships that could have appeared to influence the work reported in this paper.

## Footnotes

1 Adafruit Feather Huzzah ESP8266:https://www.adafruit.com/product/2821.

2 Pycom LoPy4:https://pycom.io/product/lopy4/.

3 Visual Studio Code IDE:https://code.visualstudio.com/.

4 LoPy4 specification:https://pycom.io/product/lopy4/ (11/01/2022)

5 Arduino MKRFOX 1200 specification:https://store.arduino.cc/products/arduino-mkr-fox-1200 (11/01/2022)

12 Docker:https://www.docker.com/ (11/01/2022)

13 Docker-compose installation:https://docs.docker.com/compose/install/ (12/01/2022)

14 Ngrok:https://ngrok.com (11/01/2022)

19 LoPy4 consumption from Pycom data-sheet:https://docs.pycom.io/gitbook/assets/specsheets/Pycom_002_Specsheets_LoPy4_v2.pdf (25/5/2022)

20 SiPy consumption from Pycom data-sheet:https://docs.pycom.io/gitbook/assets/specsheets/Pycom_002_Specsheets_SiPy_v2.pdf (25/05/2022)

21 ESP32 consumption from Espressif data-sheet:https://www.espressif.com/sites/default/files/documentation/esp32_datasheet_en.pdf (25/05/2022)

22 Precious Plastic website:https://preciousplastic.com/index.html (01/09/2022)

## Footnotes

6 The Thing Network:https://www.thethingsnetwork.org/ (11/01/2022)

7 ChirpStack website:https://www.chirpstack.io/ (11/01/2022)

8 Pybytes: https://pybytes.pycom.io/ (11/01/2022)

9 InfluxDB:https://www.influxdata.com/ (11/02/2022)

10 Grafana:https://grafana.com/ (11/02/2022)

11 Node-RED:https://nodered.org/ (11/02/2022)

15 Pycom Pymakr plugin:https://pycom.io/products/supported-networks/pymakr/ (11/01/2022)

16 Pycom Firmware Updater:https://pycom.io/downloads/#firmware (11/01/2022)

17 Pycom procedure to register a Sigfox device:https://docs.pycom.io/gettingstarted/registration/sigfox/ (11/01/2022)

18 Sigfox activation Page:https://buy.sigfox.com/activate (11/01/2022)
